# Responsive nanoparticles synergize with Curcumin to break the “reactive oxygen Species-Neuroinflammation” vicious cycle, enhancing traumatic brain injury outcomes

**DOI:** 10.1186/s12951-025-03251-y

**Published:** 2025-03-05

**Authors:** Xianhua Fu, Yongkang Zhang, Guojie Chen, Guangyao Mao, Jiajia Tang, Jin Xu, Yuhan Han, Honglin Chen, Lianshu Ding

**Affiliations:** 1 Department of Neurosurgery, The Affiliated Huaian NO.1 People’s Hospital of Nanjing Medical University, Huaian, Jiangsu China; 2 Department of Neurosurgery, The Affiliated Suqian First People’s Hospital of Nanjing Medical University, Suqian, China; 3 Brain Injury Center, Department of Neurosurgery, Ren Ji Hospital, Shanghai Jiao Tong University School of Medicine, Shanghai, China; 4 Department of Neurosurgery, The Affiliated Hospital of Xuzhou Medical University, Xuzhou, China; 5 Clinical Laboratory, Affiliated Taizhou People’s Hospital of Nanjing Medical University, Taizhou, Jiangsu China

**Keywords:** Traumatic brain injury, Curcumin, Reactive oxygen species, Neuroinflammation, NF-κB

## Abstract

Traumatic brain injury (TBI) disrupts oxygen homeostasis in the brain, leading to excessive reactive oxygen species (ROS) production and dysregulated antioxidant mechanisms, which fail to clear excess ROS. This ROS overload promotes the expression of pro-inflammatory genes, releasing cytokines and chemokines and creating a vicious “ROS-neuroinflammation” cycle, making it essential to break this cycle for effective TBI treatment. In this study, we developed cysteine-alanine-glutamine-lysine (CAQK) peptide-modified antioxidant nanoparticles (C-PPS/C) for co-delivery of curcumin (Cur) to modulate oxidative and neuroinflammatory disturbances after TBI. In TBI mice, C-PPS/C nanoparticles accumulated in injured brain regions, where poly (propylene sulfide)_120_ scavenged ROS, reducing oxidative stress, while Cur release further suppressed ROS and inflammation. C-PPS/C nanoparticles broke the “ROS-neuroinflammation” cycle, protecting the blood-brain barrier (BBB), reducing acute brain edema, and promoting long-term neurological recovery. Further investigation showed that C-PPS/C nanoparticles inhibited the NF-κB pathway, reducing pro-inflammatory gene expression and mitigating inflammation, suggesting a promising approach for TBI treatment.

**Supplementary Information:**

The online version contains supplementary material available at 10.1186/s12951-025-03251-y.

## Graphical abstract

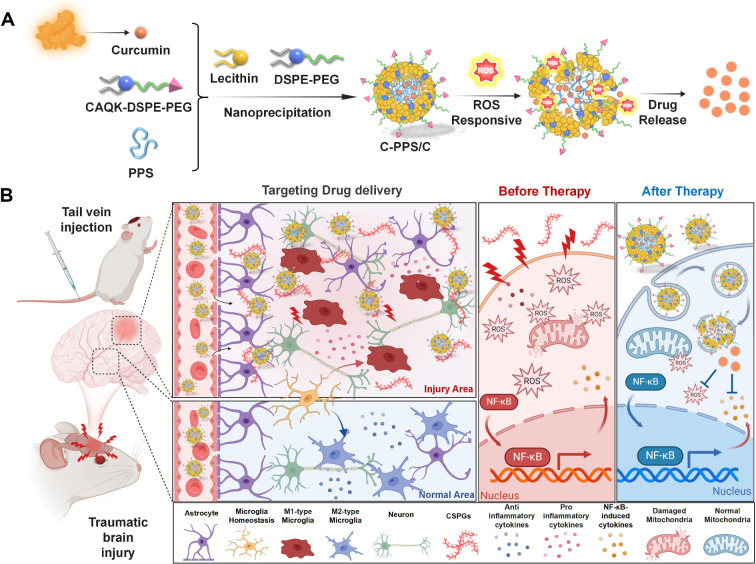

## Introduction

Traumatic brain injury (TBI) is one of the most prevalent neurological disorders, posing a significant global public health challenge. TBI results in an economic burden exceeding $400 billion annually, placing a substantial strain on economies across the globe [1, 2]. Patients with mild to moderate TBI usually recover completely within a few weeks, but some may experience long-term symptoms such as anxiety and cognitive difficulties. In contrast, severe TBI is more likely to result in long-term or permanent neurological deficits, including motor, cognitive, and behavioral impairments. The rehabilitation process for severe TBI often requires a prolonged period, and the outcomes are uncertain. TBI can be classified into primary and secondary brain injuries [3]. Primary brain injury occurs immediately after impact, directly causing damage to the brain tissue due to external forces. This type of injury is usually irreversible. Secondary brain injury develops after the initial impact and involves a series of biochemical and physiological processes that cause further brain damage. These processes can occur within hours to days following primary injury. Therefore, the treatment of TBI focuses on mitigating secondary brain injury. The main goal is to control and reduce the pathological processes associated with secondary brain injury to minimize long-term neurological deficits [4].

The brain, being highly oxygen-dependent, requires abundant blood supply to maintain normal function. The blood flow of the brain accounts for approximately 15–20% of cardiac output [5]. After TBI, ischemia and reperfusion injuries lead to oxygen supply disruption and oxidative stress, which are critical factors in secondary brain injury [6]. Post-TBI, the blood-brain barrier (BBB) is compromised, allowing harmful substances to infiltrate brain tissue and further exacerbating disruption of the oxygen supply and oxidative stress [7]. Disrupted oxygen supply and oxidative stress result in the generation of a large amount of reactive oxygen species (ROS). These ROS can initiate free radical chain reactions, causing damage to cell membranes, proteins, and DNA, thereby further compromising cell function and structure. The endogenous antioxidant mechanisms within brain tissue become dysregulated after TBI, resulting in a failure to effectively clear excessive ROS [8, 9]. Oxidative stress and inflammation interact in pathological processes following TBI, creating a vicious cycle that leads to further neural damage. ROS promote the expression of pro-inflammatory genes, increasing the release of cytokines and chemokines. During neuroinflammation, activated microglia produce more ROS, contributing to this vicious cycle of “ROS-neuroinflammation” [10, 11]. Breaking this “ROS-neuroinflammation” cycle is crucial for treating TBI. Effective strategies should focus on controlling oxidative stress and reducing inflammation to mitigate further neural damage and improve patient outcomes.

Curcumin (Cur), a natural polyphenolic compound extracted from the rhizome of turmeric, has garnered significant attention due to its diverse biological activities and potential therapeutic effects [12]. Cur has the ability to directly scavenge ROS, reducing ROS-induced cellular damage. Additionally, it can enhance the endogenous antioxidant capacity, thereby lowering oxidative stress levels. Cur can inhibit the expression and release of pro-inflammatory cytokines and chemokines, reducing inflammation [13]. Furthermore, it promotes the polarization of microglia toward the M2 phenotype, exerting anti-inflammatory and neuroprotective effects [14]. However, the poor solubility of Cur in water severely limits its distribution and absorption in the body; it also undergoes rapid metabolism in vivo, leading to low bioavailability. The stability of Cur is another challenge; it is prone to oxidation, which reduces its activity before it reaches the target area. The presence of the BBB further hinders the ability of Cur to effectively penetrate the central nervous system, limiting its therapeutic efficacy [14, 15]. Nanotechnology offers significant advantages in the application of therapies within the nerve injury [16, 17]. Firstly, nanoparticulate carriers enable targeted drug delivery to specific neural cells or injury sites, improving therapeutic efficacy and reducing systemic side effects. Additionally, nanoparticles can effectively cross the BBB, ensuring precise drug delivery to brain tissues. Nanocapsulation enhances the stability of drugs, preventing premature degradation or metabolism. Furthermore, nanotechnology allows for sustained drug release, prolonging therapeutic effects and reducing dosing frequency [18]. Therefore, we can leverage nanotechnology to overcome the limitations of Cur application and better harness its neuroprotective potential.

**Fig. 1:**
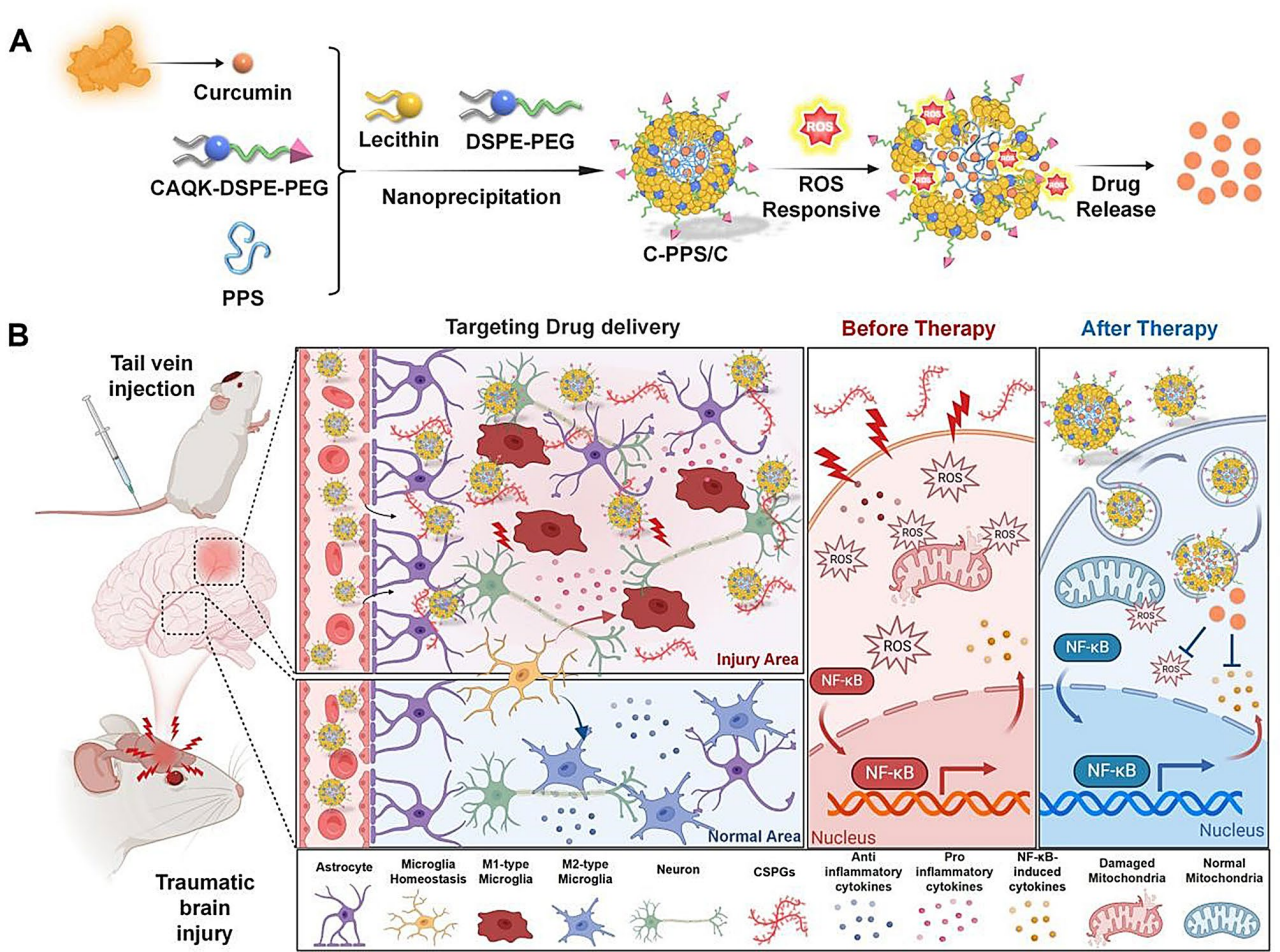
Schematic of C-PPS/C nanoparticles for TBI treatment. (**A**) The preparation process of C-PPS/C nanoparticles involves designing a system that can release curcumin in response to ROS (reactive oxygen species). (**B**) After TBI, C-PPS/C is administered via tail vein injection. C-PPS/C nanoparticles bind to the high levels of CSPGs in the injury site, allowing it to selectively accumulate in the damaged brain tissue. In response to ROS, C-PPS/C nanoparticles consume ROS while simultaneously releasing curcumin, reducing oxidative stress, alleviating neuroinflammation, and providing neuroprotection

In this study, we developed cysteine-alanine-glutamine-lysine (CAQK) peptide-modified nanoparticles for the delivery of Cur (C-PPS/C) to treat TBI (Fig. 1). The CAQK peptide modification of C-PPS/C facilitates nanoparticle penetration of the BBB and recognition of the increased expression of chondroitin sulfate proteoglycans in injured brain tissue, thus promoting accumulation at the injury site. The hydrophobic core of C-PPS/C, which is composed of poly (propylene sulfide) (PPS), acts as a thioether that can react with and scavenge ROS. Upon accumulation in injured brain tissue, the PPS core of the C-PPS/C nanoparticles responds to and depletes ROS while simultaneously releasing Cur to exert neuroprotective effects. We propose that C-PPS/C breaks the vicious cycle of “ROS-neuroinflammation” following TBI, thereby modulating the oxidative and inflammatory microenvironment and improving TBI outcomes. This novel approach presents a potential therapeutic strategy for the treatment of TBI.

## Results

### Preparation and characterization of C-PPS/C

**Fig. 2:**
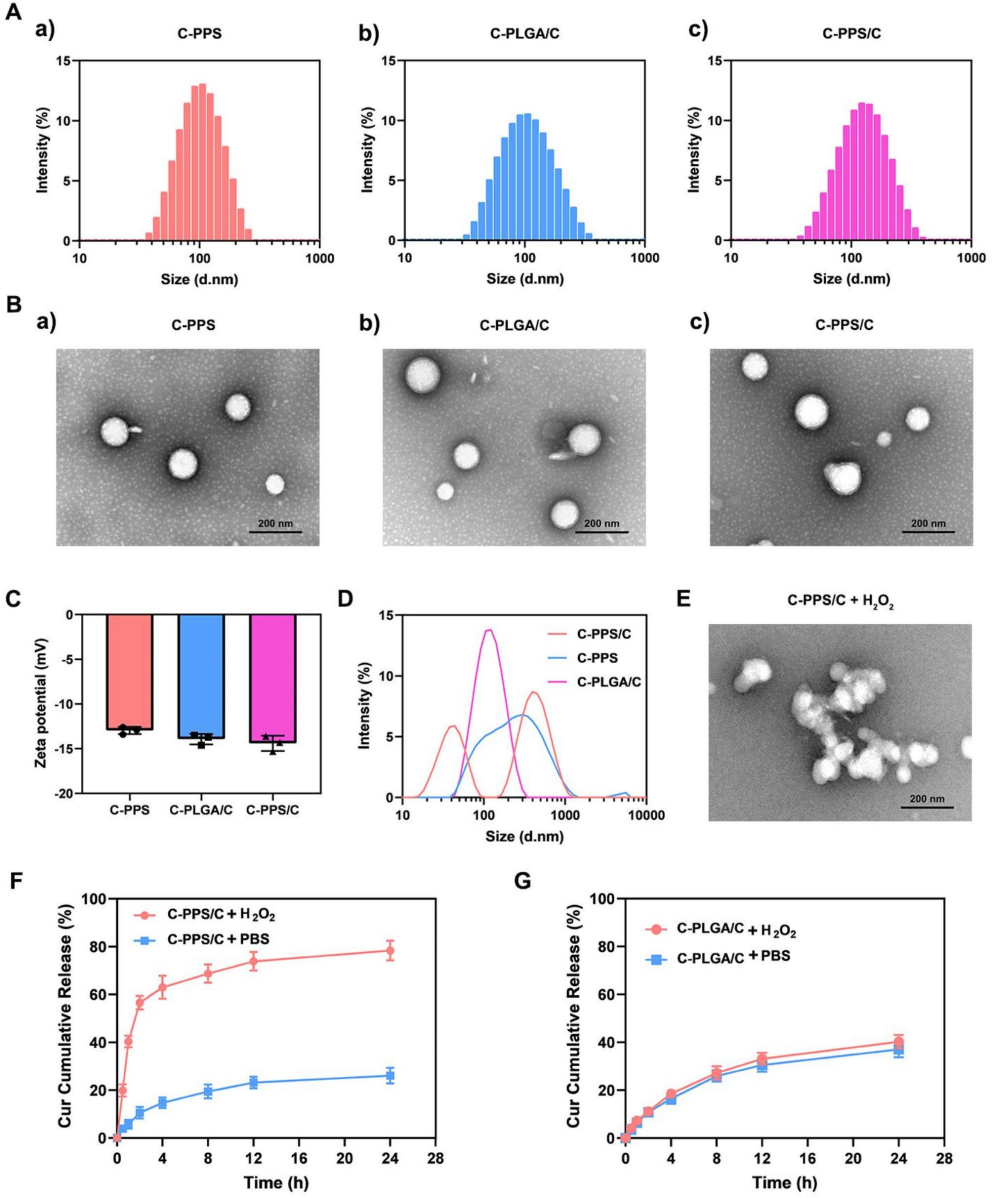
Preparation and characterization of nanoparticles. (**A**) Size distribution of (a) C-PPS, (b) C-PLGA/C and (c) C-PPS/C nanoparticles. (**B**) TEM image of (a) C-PPS, (b) C-PLGA/C and (c) C-PPS/C nanoparticles. Scale bar, 200 nm. (**C**) Zeta potentials of C-PPS, C-PLGA/C and C-PPS/C nanoparticles. (**D**) Size distribution of nanoparticles in H_2_O_2_. (**E**) TEM image of C-PPS/C nanoparticles in H_2_O_2_. (**F**) The drug release efficiency of Cur from C-PPS/C nanoparticles in different environments (PBS and H_2_O_2_) in vitro. *n* = 3. (**G**) The drug release efficiency of Cur from C-PLGA/C nanoparticles in different environments (PBS and H_2_O_2_) in vitro. *n* = 3. Data are presented as the means ± SD

The aim of this study was to develop nanoparticles with the ability to scavenge ROS and deliver Cur. First, hydrophobic PPS_120_ was synthesized using reversible addition-fragmentation chain transfer polymerization. Under the action of ROS, the hydrophobic polysulfide units in PPS are converted into hydrophilic polysulfoxide units [19]. During the degradation of PPS, ROS in the microenvironment were consumed. Thus, PPS_120_ serves as the hydrophobic core of the nanoparticles, exhibiting ROS-responsive characteristics and the ability to scavenge ROS. The structure of the synthesized PPS_120_ was confirmed by ^1^H NMR (Fig. S1). Nanoparticles targeting the injured area post-TBI (C-PPS) were formed through single-step nanoprecipitation self-assembly using PPS_120_, DSPE-PEG_2000_, DSPE-PEG_2000_-CAQK, and lecithin. These nanoparticles were designed for the delivery of Cur (C-PPS/C). Additionally, nanoparticles with a poly (lactic-co-glycolic acid) (PLGA) hydrophobic core loaded with Cur (C-PLGA/C) were prepared as a control group for subsequent experiments. The average diameter of C-PPS was 99.6 ± 2.57 nm, that of C-PLGA/C was 108.1 ± 4.06 nm, and that of C-PPS/C was 115.17 ± 3.06 nm (Fig. 2A). Transmission electron microscopy (TEM) images revealed that the C-PPS, C-PLGA/C, and C-PPS/C nanoparticles were spherical, exhibited good monodispersity, and were relatively uniform (Fig. 2B). The zeta potentials of C-PPS, C-PLGA/C and C-PPS/C nanoparticles were − 12.96 ± 0.4 mV, -13.93 ± 0.59 mV and − 14.4 ± 0.85 mV, respectively (Fig. 2C).

The key aspect of synthesized nanoparticles is to assess their responsiveness to ROS. We conducted several experiments to verify their responsiveness to ROS and their ability to release Cur. We added H_2_O_2_ to solutions of C-PPS, C-PLGA/C, and C-PPS/C nanoparticles and measured their particle sizes. We observed that the particle sizes of the C-PPS and C-PPS/C groups increased, displaying bimodal peaks, whereas those of the C-PLGA/C group remained stable due to the inability of PLGA to respond to H_2_O_2_ (Fig. 2D). Transmission electron microscopy observations of C-PPS/C nanoparticles in the presence of H_2_O_2_ revealed that the spherical nanostructures had changed, with the nanoparticles decomposing. These findings indicate that nanoparticles prepared with PPS as the hydrophobic core can respond to ROS, leading to their degradation. As shown in Fig. 2F and G, we used high performance liquid chromatography (HPLC) to detect the Cur release capability of C-PPS/C and C-PLGA/C nanoparticles in the presence and absence of H_2_O_2_. Due to the responsiveness of PPS to H_2_O_2_, C-PPS/C nanoparticles can release Cur more rapidly in the presence of H_2_O_2_ but exhibit a lower drug release efficiency in the absence of H_2_O_2_. However, C-PLGA/C nanoparticles, which lack ROS responsiveness because of the presence of PLGA, consistently exhibit a lower drug release efficiency under both conditions.

### C-PPS/C nanoparticles have antioxidant effects in vitro

**Fig. 3:**
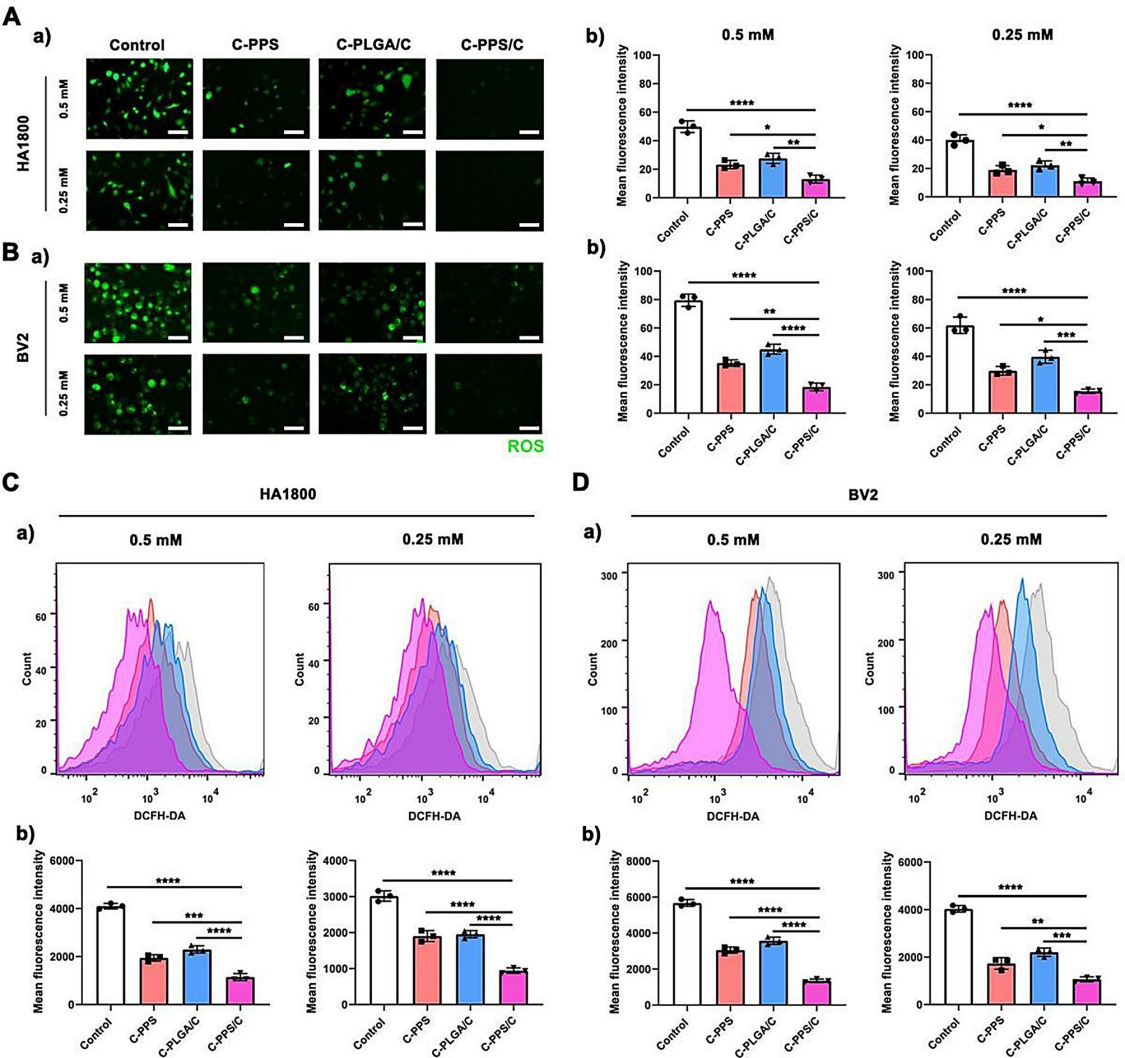
C-PPS/C nanoparticles have antioxidant effects in vitro. (**A**) a) ROS production in HA1800 with 0.5 mM and 0.25 mM H_2_O_2_. Scale bar = 100 μm. b) Quantitative mean fluorescence intensity in each group. *n* = 3. (**B**) (a) ROS production in BV2 with 0.5 mM and 0.25 mM H_2_O_2_. Scale bar = 100 μm. (b) Quantitative mean fluorescence intensity in each group. *n* = 3. (**C**) (a) ROS production in HA1800 with 0.5 mM and 0.25 mM H_2_O_2_ are indicated with flow cytometry. (b) Quantitative mean fluorescence intensity in each group. *n* = 3. (**D**) (a) ROS production in BV2 with 0.5 mM and 0.25 mM H_2_O_2_ are indicated with flow cytometry. (b) Quantitative mean fluorescence intensity in each group. *n* = 3. Data are presented as the means ± SD. ^**^p < 0.01, ^***^p < 0.001 and ^****^p < 0.0001

We further validated the ability of C-PPS/C nanoparticles to scavenge ROS and inhibit oxidative stress through cell-level experiments. Two neural cell lines, human astrocyte 1800 (HA1800) and a microglial cell line (BV2), were used to assess the antioxidant capacity of the nanoparticles following H_2_O_2_-induced injury (0.25 mM, 0.5 mM). Levels of ROS were analyzed using fluorescent probes (Fig. 3A and B) and flow cytometry (Fig. 3C and D). The C-PPS group exhibited notable antioxidant capacity due to the polysulfide units in PPS reacting with H_2_O_2_, transforming into hydrophilic polysulfoxide units and directly consuming H_2_O_2_, thereby reducing oxidative stress at its source (C-PPS vs. C-PPS/C; Fig. 3Ab 0.5 mM, *p* = 0.0252, 95%CI: 1.358 to 18.98; Fig. 3Ab 0.25 mM, *p* = 0.0465, 95% CI: 0.127 to 15.94; Fig. Bb 0.5 mM, *p* = 0.0011, 95% CI: 8.246 to 25.29; Fig. Bb 0.25 mM, *p* = 0.0106, 95% CI: 3.766 to 24.97; Fig. 3Cb 0.5 mM, *p* = 0.0005, 95% CI: 439 to 1160; Fig. 3Cb 0.25 mM, *p* < 0.0001, 95% CI: 639.6 to 1277; Fig. Db 0.5 mM, *p* < 0.0001, 95% CI: 1261 to 2142; Fig. 3Db 0.25 mM, *p* < 0.0068, 95% CI: 210 to 1118). The C-PLGA/C group also presented some antioxidant effects attributable to the presence of Cur, which can reduce ROS levels to a certain extent (C-PLGA/C vs. C-PPS/C; Fig. 3Ab 0.5 mM, *p* = 0.0033, 95% CI: 5.691 to 23.31; Fig. 3Ab 0.25 mM, *p* = 0.0077, 95% CI: 3.427 to 19.24; Fig. Bb 0.5 mM, *p* < 0.0001, 95% CI: 17.98 to 35.02; Fig. Bb 0.25 mM, *p* = 0.0004, 95% CI: 13.67 to 34.87; Fig. 3Cb 0.5 mM, *p* < 0.0001, 95% CI: 790.4 to 1511; Fig. 3Cb 0.25 mM, *p* < 0.0001, 95% CI: 690.6 to 1328; Fig. 3Db 0.5 mM, *p* < 0.0001, 95% CI: 1780 to 2661; Fig. 3Db 0.25 mM, *p* = 0.0002, 95% CI: 684.7 to 1593). Importantly, the C-PPS/C nanoparticles demonstrated the best antioxidant capacity; this occurred because C-PPS/C nanoparticles not only directly consume H_2_O_2_ through PPS but also release encapsulated Cur within PPS, enhancing antioxidant capacity and further reducing oxidative stress levels in cells post-H_2_O_2_ injury. However, in clinical scenarios, the oxidative stress process following TBI is more severe and complex. Therefore, single-cell level assays are insufficient. Consequently, subsequent experiments will further verify the antioxidant capacity at the animal level.

### Targeted delivery of C-PPS/C nanoparticles to the TBI site

**Fig. 4:**
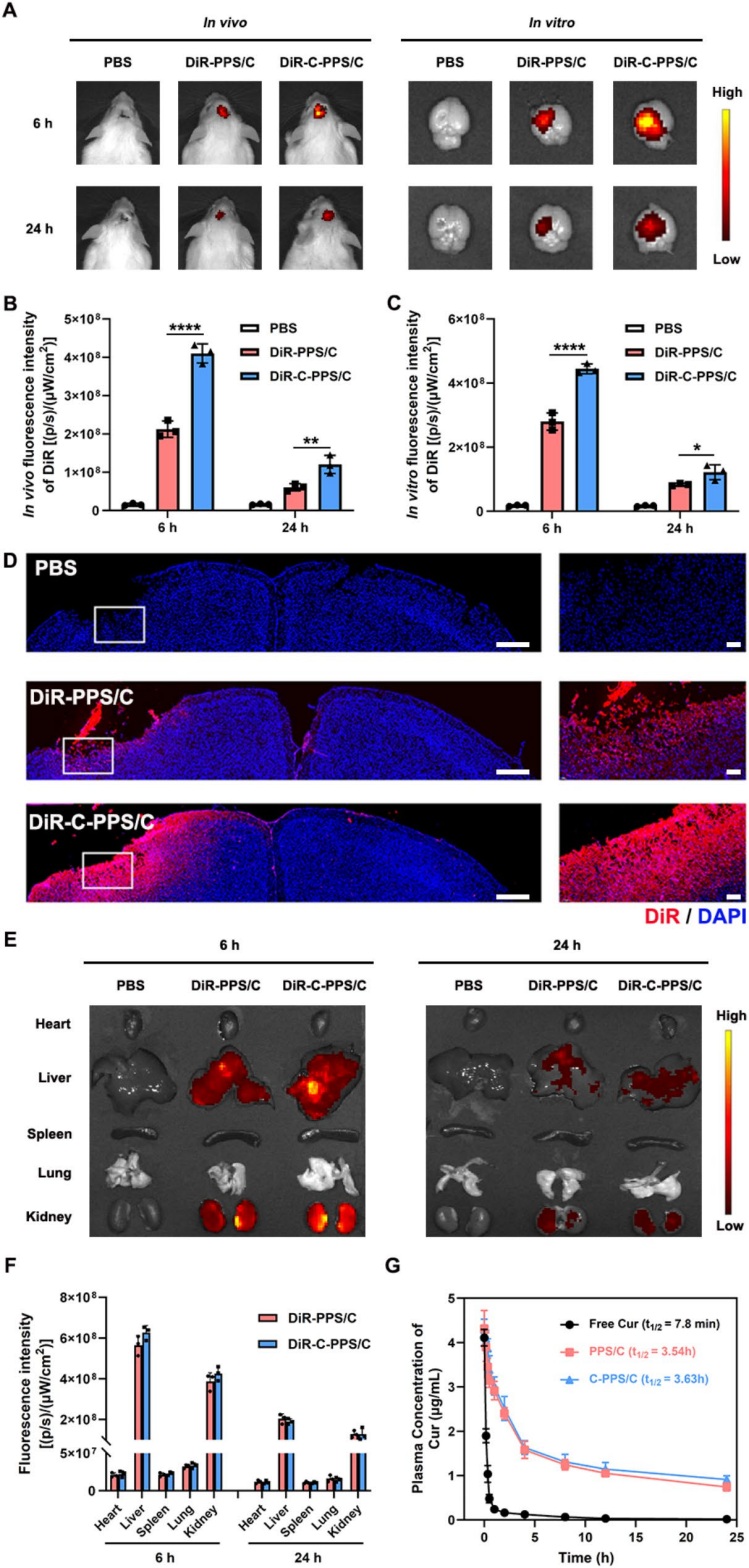
Pharmacokinetics, targeting and metabolic profile of C-PPS/C nanoparticles. (**A**) The DiR fluorescence images of TBI mice after injection of PBS, DiR-PPS/C and DiR-C-PPS/C nanoparticles to observe their targeting injury region ability. Quantitative analysis of fluorescence intensity in vivo (**B**) and in vitro (**C**). (**D**) Brain distribution of PBS, DiR-PPS/C and DiR-C-PPS/C nanoparticles. scale bar = 100 μm. (**E**) The DiR fluorescence images of major organs after i.v. injection of PBS, DiR-PPS/C and DiR-C-PPS/C. (**F**) Quantification of DiR accumulation in major organs. *n* = 3. (**G**) In vivo pharmacokinetics of Free Cur, PPS/C and C-PPS/C in mice. Data are presented as the means ± SD. ^*^p < 0.05, ^**^p < 0.01, ^****^p < 0.0001

To effectively exert the neuroprotective effects of Cur, it is crucial to achieve high concentrations of Cur at the site after TBI. However, the BBB acts as a selective barrier, preventing Cur from entering brain tissue from the blood. According to previous studies, CAQK is a tetrapeptide that can target chondroitin sulfate proteoglycans at the injury site following TBI, delivering drugs to the brain injury site [20, 21]. Therefore, C-PPS nanoparticles were prepared as drug carriers. These nanoparticles can effectively cross the BBB and, due to surface modification with CAQK, target the injury are after TBI. This targeting allows for the enrichment of the drug at the trauma site, thereby enhancing its therapeutic effect. Therefore, to evaluate the targeting effect, we intravenously injected 1,1’-dioctadecyl-3,3,3’,3-tetramethylin-dotricarbocyanine iodide (DiR)-conjugated nanoparticles were introduced into mice via the tail vein after TBI. We constructed two types of nanoparticles: PPS/C nanoparticles without CAQK modification (DiRi-PPS/C) and C-PPS/C nanoparticles with CAQK modification (DiR-C-PPS/C). Six and 24 h post-injection, observations were made using an small animal in vivo imaging system. As shown in Fig. 4A-C, at both 6 and 24 h post-injection, the DiR fluorescence intensity in the brains of the mice treated with DiR-C-PPS/C nanoparticles was greater than that in the brains of the mice treated with DiR-PPS/C nanoparticles (DiR-PPS/C vs. DiR-C-PPS/C; Fig. 4B 6 h, *p* < 0.0001, 95% CI: -245154450 to -150178883; Fig. 4B 2^4^h, *p* = 0.006, 95% CI: -96430994 to -22902339; Fig. 4C 6 h, *p* < 0.0001, 95% CI: -209747298 to -119586035; Fig. 4C 2^4^h, *p* = 0.0327, 95% CI: -72664878 to -3935122). To further verify whether the nanoparticles can accumulate in the injury region, we isolated brain tissue from mice 24 h after drug injection and performed histological analysis. We observed that the CAQK-modified nanoparticles accumulated better in the injured area (Fig. 4D). These results indicate that the CAQK-modified nanoparticles can effectively cross the BBB and target the brain injury area.

Analysis of the distribution of nanoparticles in the heart, liver, spleen, lungs, and kidneys revealed that the nanoparticles were metabolized primarily by the liver and kidneys. Due to the targeting effect of CAQK, DiR-C-PPS/C nanoparticles accumulated more in the brain tissue, resulting in a lower distribution in the liver and kidneys than DiR-PPS/C nanoparticles (Fig. 4E and F). Analysis of drug concentrations in serum revealed that free Cur was cleared more rapidly, while its formulation into nanoparticles increased the drug’s half-life, thereby extending its duration of action. Moreover, CAQK modification did not alter the half-life of the nanoparticles (Fig. 4G).

### C-PPS/C nanoparticles have good biocompatibility

Biocompatibility is a crucial factor influencing the clinical application of a material. As shown in Fig. S2A, on the 7th day post-injury, biocompatibility was evaluated through histopathological examination of organs (heart, liver, spleen, lung, and kidney) and blood tests of tissues from each group of mice. No significant pathological changes were observed in any group. The levels of aspartate aminotransferase (AST), alanine aminotransferase (ALT), creatinine (CREA), and blood urea nitrogen (BUN) in each group were within normal ranges (Fig. 2SB-E). These results indicate that the nanoparticles have good biocompatibility.

### C-PPS/C nanoparticles reduce brain edema and protect the BBB after TBI in vivo

**Fig. 5:**
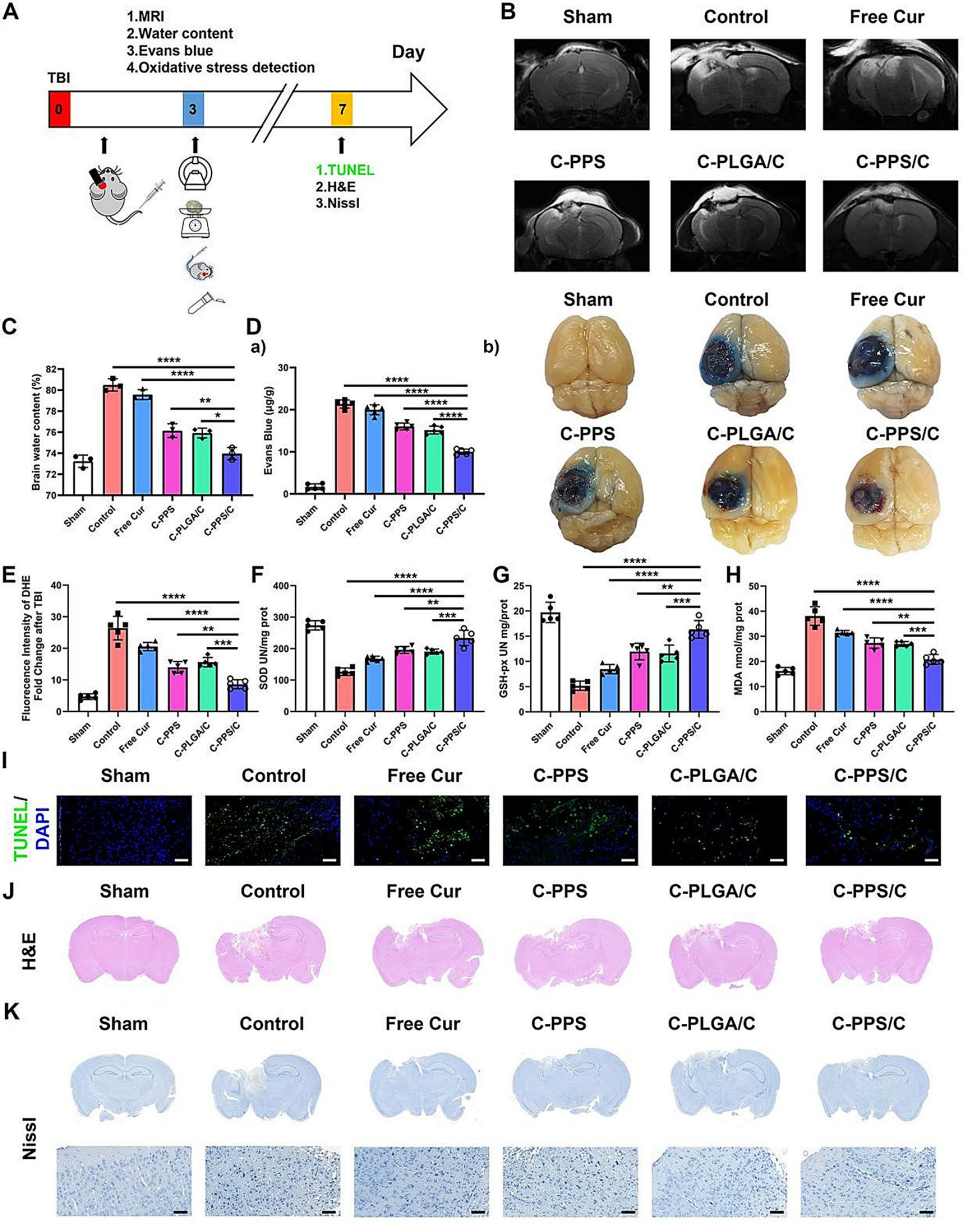
C-PPS/C nanoparticles reduce brain edema and protect BBB after TBI. (**A**) Schematic diagram of TBI model experiments. (**B**) Representative T2-weighted images of each group. (**C**) Water content in brain tissue of each group. (**D**) (a) Quantification of Evans Blue leakage of each group. (b) Representative Evans Blue leakage images of each group. *n* = 5. C-PPS/C nanoparticles have antioxdant effects *in vivo.* (**E**) Fluorescence intensity of DHE fold change of each group. *n* = 5. (**F**) SOD, (**G**) GSH-px and (**H**) MDA of each group. *n* = 5. C-PPS/C nanoparticles have neuroprotective effects *in vivo.* (**I**) Representative images of TUNEL staining on the 7th day after TBI. Scale bar = 50 μm. (**J**) Sagittal H&E brain staining. (**K**) Nissl brain staining. Scale bar = 50 μm. Data are presented as the means ± SD. ^*^p < 0.05, ^**^p < 0.01, ^***^p < 0.001 and ^****^p < 0.0001

Disruption of the BBB and the resulting brain edema are two interrelated pathological processes that significantly impact the progression of TBI. When the BBB is damaged, plasma constituents such as water and proteins can infiltrate brain tissue, leading to fluid accumulation and subsequent brain edema. This edema increases intracranial pressure, which in turn exacerbates BBB damage [7, 22, 23]. Elevated intracranial pressure compresses and deforms endothelial cells, further impairing the integrity of the BBB and creating a vicious cycle [24]. Protecting and restoring the integrity of the BBB can effectively reduce the transmission of fluid from the vasculature into brain tissue, thereby alleviating brain edema [25]. Consequently, safeguarding the BBB is a critical strategy in the treatment of cerebral edema [26]. Therefore, protecting the BBB and breaking the “BBB disruption-brain edema” cycle constitute crucial strategies in the treatment of brain edema. In this study, we conducted several experiments to demonstrate whether C-PPS/C nanoparticles can improve cerebral edema following traumatic brain injury by protecting the BBB. The diagram and schedule are shown in Fig. 5A. As shown in Fig. 5B and Fig. S3, the extent of brain edema in the injured brain tissue of mice after TBI was observed using the T2 sequence of magnetic resonance imaging (MRI). Mice treated with C-PPS and C-PLGA/C nanoparticles exhibited varying degrees of improvement in brain edema, with the C-PPS/C group showing the least edema (C-PPS vs. C-PPS/C, *p* = 0.0169, 95% CI: 0.4923 to 6.776; C-PLGA/C vs. C-PPS/C, *p* = 0.0438, 95% CI: 0.06226 to 6.346). The same trend was observed in the water content experiment (Fig. 5C) (C-PPS vs. C-PPS/C, *p* = 0.0047, 95% CI: 0.6455 to 3.720; C-PLGA/C vs. C-PPS/C, *p* = 0.0105, 95% CI: 0.4245 to 3.499). We evaluated the integrity of the BBB in each group of mice using the Evans blue assay (Fig. 5D). Notably, C-PPS/C nanoparticles exhibited the best therapeutic effect, with less BBB damage and correspondingly milder brain edema (Control vs. C-PPS/C, *p* < 0.0001, 95% CI: 9.609 to 13.06; Free Cur vs. C-PPS/C, *p* < 0.0001, 95% CI: 8.228 to 11.68; C-PPS vs. C-PPS/C, *p* < 0.0001, 95% CI: 4.278 to 7.729; C-PLGA/C vs. C-PPS/C, *p* < 0.0001, 95% CI: 3.403 to 6.854). This phenomenon indicates that C-PPS/C nanoparticles combine the antioxidant properties of PPS with the neuroinflammatory inhibition of Cur. By improving oxygenation and the inflammatory microenvironment after TBI, this combination breaks the “BBB disruption-brain edema” vicious cycle, protects the BBB, and alleviates brain edema.

### C-PPS/C nanoparticles have antioxidant effects in vivo

Based on the antioxidant capacity demonstrated by C-PPS/C nanoparticles in vitro, we conducted a series of in vivo experiments to further verify its ability to inhibit excessive oxidative stress following TBI. As shown in Fig. 5E, on the 3rd day post-TBI, we utilized dihydroethidium (DHE) staining to detect superoxide levels in the mouse brain. We found that the superoxide levels in the C-PPS, C-PLGA/C, and C-PPS/C groups decreased to varying degrees, with the C-PPS/C group showing a significant reduction in DHE levels (Control vs. C-PPS/C, *p* < 0.0001, 95% CI: 13.88 to 21.69; Free Cur vs. C-PPS/C, *p* < 0.0001, 95% CI: 8.037 to 15.85; C-PPS vs. C-PPS/C, *p* = 0.0034, 95% CI: 1.463 to 9.273; C-PLGA/C vs. C-PPS/C, *p* = 0.0001, 95% CI: 3.075 to 10.89). We further assessed the endogenous antioxidant levels and were pleasantly surprised that, in addition to the inherent ROS scavenging capability, they also enhanced the brain’s own antioxidant capacity. The levels of superoxide dismutase (SOD), glutathione peroxidase (GSH-px), and malondialdehyde (MDA) serve as indicators of antioxidant activity in the brain: SOD eliminates the initiators of free radical reactions, GSH-px interrupts the lipid peroxidation chain reaction, and MDA, as the end product of oxidative stress, reflects the extent of damage caused by ROS in brain (Fig. 5F-H). Cur and PPS not only directly react with and eliminate ROS but also enhance the endogenous antioxidant capacity of brain tissue; this led to increased levels of SOD and GSH-px and decreased levels of MDA in the C-PPS and C-PLGA/C groups. The C-PPS/C group, combining the advantages of both PPS and Cur, demonstrated even more greater antioxidant capacity (Fig. 5F Control vs. C-PPS/C, *p* < 0.0001, 95% CI: -135.0 to -79.86; Free Cur vs. C-PPS/C, *p* < 0.0001, 95% CI: -93.97 to -38.83; C-PPS vs. C-PPS/C, *p* = 0.0034, 95% CI: -65.45 to -10.31; C-PLGA/C vs. C-PPS/C, *p* = 0.0007, 95% CI: -70.95 to -15.81; Fig. 5G Control vs. C-PPS/C, *p* < 0.0001, 95% CI: -14.19 to -8.162; Free Cur vs. C-PPS/C, *p* < 0.0001, 95% CI: -10.90 to -4.878; C-PPS vs. C-PPS/C, *p* = 0.0017, 95% CI: -7.418 to -1.394; C-PLGA/C vs. C-PPS/C, *p* = 0.0007, 95% CI: -7.780 to -1.756; Fig. 5H Control vs. C-PPS/C, *p* < 0.0001, 95% CI: 13.21 to 21.33; Free Cur vs. C-PPS/C, *p* < 0.0001, 95% CI: 6.615 to 14.74; C-PPS vs. C-PPS/C, *p* = 0.0005, 95% CI: 2.503 to 10.63; C-PLGA/C vs. C-PPS/C, *p* = 0.0011, 95% CI: 2.103 to 10.23).

### C-PPS/C nanoparticles have neuroprotective effects in vivo

The most direct impact of TBI on the brain is neuronal cell death, which can lead to severe consequences. To assess the neuroprotective effect of C-PPS/C nanoparticles, we examined neuronal cells in brain tissue sections. As shown in Fig. 5I, Terminal deoxynucleotidyl transferase-mediated dUTP nick end labeling (TUNEL) staining revealed a high level of neuronal apoptosis in the injured brain tissue of the Control group. While the Free Cur group showed some improvement in reducing apoptosis, the lack of targeting resulted in less effective treatment compared with that in the C-PLGA /C group. The C-PPS group exhibited reduced apoptosis due to the scavenging of ROS post-TBI, which decreased lipid peroxidation, protein oxidation, and DNA damage. C-PPS/C nanoparticles, combining the advantages of both PPS and Cur and targeting damaged brain tissue, demonstrated the lowest TUNEL levels in injured tissue afterTBI.

We observed brain tissue and neuronal damage in each group of mice via hematoxylin and eosin (H&E) and Nissl staining. Hematoxylin and eosin staining images revealed that the brain tissue defects in the mice treated with nanomedicine were less severe than those in the Control group, with the C-PPS/C group showing the least amount of tissue damage (Fig. 5J). Nissl staining provided a direct indication of neuronal damage. In the Sham group, numerous normal Nissl bodies were observed, whereas in the Control group, Nissl bodies were significantly reduced, with injured neurons exhibiting shrunken cell bodies, condensed or displaced nuclei, and irregular cell outlines. Post-treatment, an increased number of normal Nissl bodies were observed in the damaged areas, indicating a greater presence of healthy neurons. The number and structure of Nissl bodies in the C-PPS/C group were similar to those in the Sham group, suggesting that C-PPS/C effectively protects neurons and mitigates the severity of the pathological changes (Fig. 5K).

### C-PPS/C nanoparticles have anti-neuroinflammatory effects in vivo

**Fig. 6:**
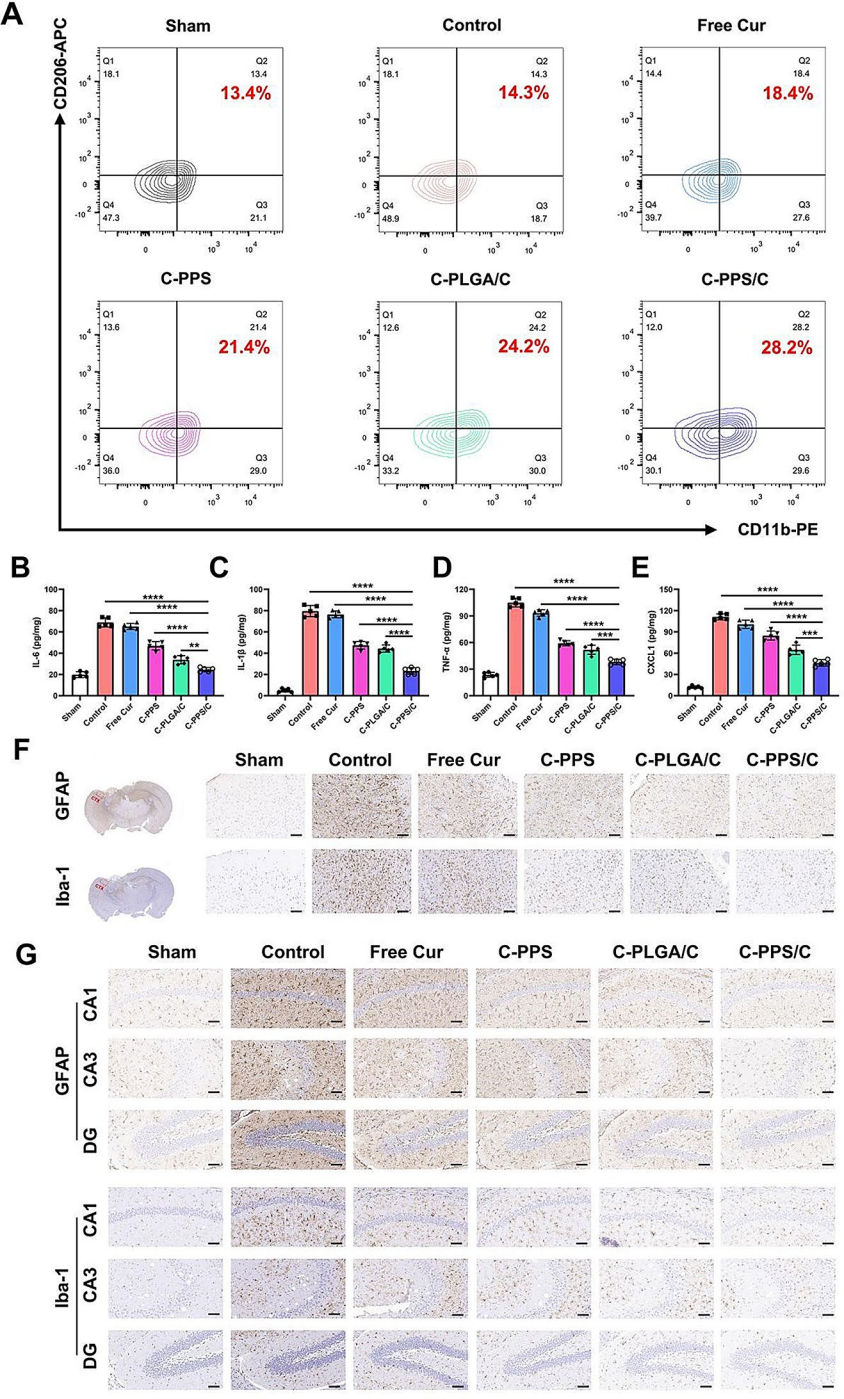
C-PPS/C nanoparticles have neuroprotective and anti-inflammatory effects *in vivo.* (**A**) Detection of macrophage type in brain tissue of each group. *n* = 3. (**B**) IL-6, (**C**), IL-1β, (**D**) TNF-α, and (**E**) CXCL-1 levels of each group. *n* = 5. (**F**) Representative images of GFAP and Iba-1 staining of the injured side of the cerebral cortex (CTX) of each group. Scale bar = 50 μm. (**G**) Representative images of GFAP and Iba-1 staining of the hippocampal area of each group. Scale bar = 50 μm. Data are presented as the means ± SD. ^**^p < 0.01, ^***^p < 0.001 and ^****^p < 0.0001

The neuroinflammatory response following TBI plays a critical role in brain tissue damage. Neuroinflammation is the repose of the central nervous system to injury or pathological conditions, and involves various cell types [11]. Microglia, the primary immune cells of the central nervous system, play an important role in neuroinflammation [27, 28]. Following TBI, microglia are rapidly activated and migrate to the injury site, where they release a variety of inflammatory mediators such as cytokines (e.g., interleukin-6 (IL-6), interleukin-1β (IL-1β)), tumor necrosis factor-α (TNF-α)), and chemokines (e.g., C-X-C ligand-1 (CXCL-1)). These mediators are crucial in the local inflammatory response, influencing neurons and other glial cells [29]. Microglia can be activated into different states after TBI [30]. M2-type microglia are essential for the resolution of inflammation and tissue repair, assisting in the restoration of neural function and protecting neurons. By modulating the polarization state of microglia and promoting their shift towards the M2 phenotype, the release of inflammatory mediators can be reduced, neuronal damage can be minimized, and neuroregeneration can be facilitated [31, 32]. Using flow cytometry to detect M2-type microglia in brain tissue post-TBI, we found that PPS and Cur increased the number of M2-type microglia in TBI mice (Fig. 6A and Fig. S4) (Control vs. C-PPS/C, *p* < 0.0001, 95% CI: -18.13 to -11.00; Free Cur vs.C-PPS/C, *p* < 0.0001, 95% CI: -13.53 to -6.405; C-PPS vs. C-PPS/C, *p* = 0.0006, 95% CI: -9.962 to -2.838; C-PLGA/C vs. C-PPS/C, *p* = 0.0096, 95% CI: -8.162 to -1.038). Furthermore, by measuring cytokines and chemokines, we observed that neuroinflammation in TBI mice treated with nanomedicine was correspondingly inhibited (Fig. 6B-E) (Fig. 6B Control vs. C-PPS/C, *p* < 0.0001, 95% CI: 37.74 to 51.03; Free Cur vs. C-PPS/C, *p* < 0.0001, 95% CI: 33.90 to 47.19; C-PPS vs. C-PPS/C, *p* < 0.0001, 95% CI: 15.72 to 29.00; C-PLGA/C vs. C-PPS/C, *p* = 0.0028, 95% CI: 2.643 to 15.93; Fig. 6C Control vs. C-PPS/C, *p* < 0.0001, 95% CI: 49.40 to 63.08; Free Cur vs. C-PPS/C, *p* < 0.0001, 95% CI: 46.24 to 59.92; C-PPS vs. C-PPS/C, *p* < 0.0001, 95% CI: 17.26 to 30.95; C-PLGA/C vs. C-PPS/C, *p* < 0.0001, 95% CI: 14.12 to 27.81; Fig. 6D Control vs. C-PPS/C, *p* < 0.0001, 95% CI: 59.37 to 74.42; Free Cur vs. C-PPS/C, *p* < 0.0001, 95% CI: 47.69 to 62.74; C-PPS vs. C-PPS/C, *p* < 0.0001, 95% CI: 13.51 to 28.57; C-PLGA/C vs. C-PPS/C, *p* = 0.0001, 95% CI: 6.186 to 21.24; Fig. 6E Control vs. C-PPS/C, *p* < 0.0001, 95% CI: 54.74 to 74.95; Free Cur vs.C-PPS/C, *p* < 0.0001, 95% CI: 44.34 to 64.55; C-PPS vs. C-PPS/C, *p* < 0.0001, 95% CI: 28.38 to 48.60; C-PLGA/C vs. C-PPS/C, *p* = 0.0001, 95% CI: 8.337 to 28.55). The C-PPS/C group presented the highest levels of M2-type microglia and the lowest levels of inflammatory cytokines and chemokines. This is attributed to the C-PPS/C nanoparticles combining the direct antioxidant effects of PPS with the excellent anti-inflammatory and antioxidant properties of Cur. C-PPS/C nanoparticles modulate the post-TBI oxygen and inflammatory microenvironment, thereby providing superior neuroprotection.

In the neuroinflammatory response, glial fibrillary acid protein (GFAP) and ionized calcium-binding adapter molecule 1(Iba-1) are two crucial markers used to identify the activation states of astrocyte and microglia, respectively [33, 34]. By simultaneously detecting GFAP and Iba-1, it is possible to comprehensively assess the activation states of microglia and astrocytes, providing insight into the neuroinflammatory response in the central nervous system following TBI. We assessed the expression of GFAP and Iba-1 in the brain tissue of mice following TBI using immunohistochemistry (Fig. 6F). By examining the expression of GFAP and Iba-1 in the cortical injury sites, we observed that the levels were reduced to varying degrees in the C-PPS and C-PLGA/Cur groups, indicating that the nanomedicine delivered to the brain injury site exerted anti-inflammatory effects. The C-PPS/C group presented the lowest expression levels of GFAP and Iba-1, demonstrating the most effective therapeutic outcome.

The hippocampus plays a critical role in various cognitive functions and emotional regulation; it is a central hub for episodic and spatial memory. Additionally, the hippocampus is involved in emotion processing and regulation, particularly in relation to stress responses and emotional memory. Following trauma, the inflammatory response in the hippocampus intensifies, and degenerative changes in this region are closely associated with the prognosis of TBI. As shown in Fig. 6G, we observed the expression levels of GFAP and Iba-1 in the Cornu Ammonis 1 (CA1), Cornu Ammonis 3 (CA3), and Dentate Gyrus (DG) regions of the hippocampus across different groups. We found a similar trend to that observed in the injured cortex.

### RNA-sequencing analysis of inflammation changes following C-PPS/C treatment

**Fig. 7:**
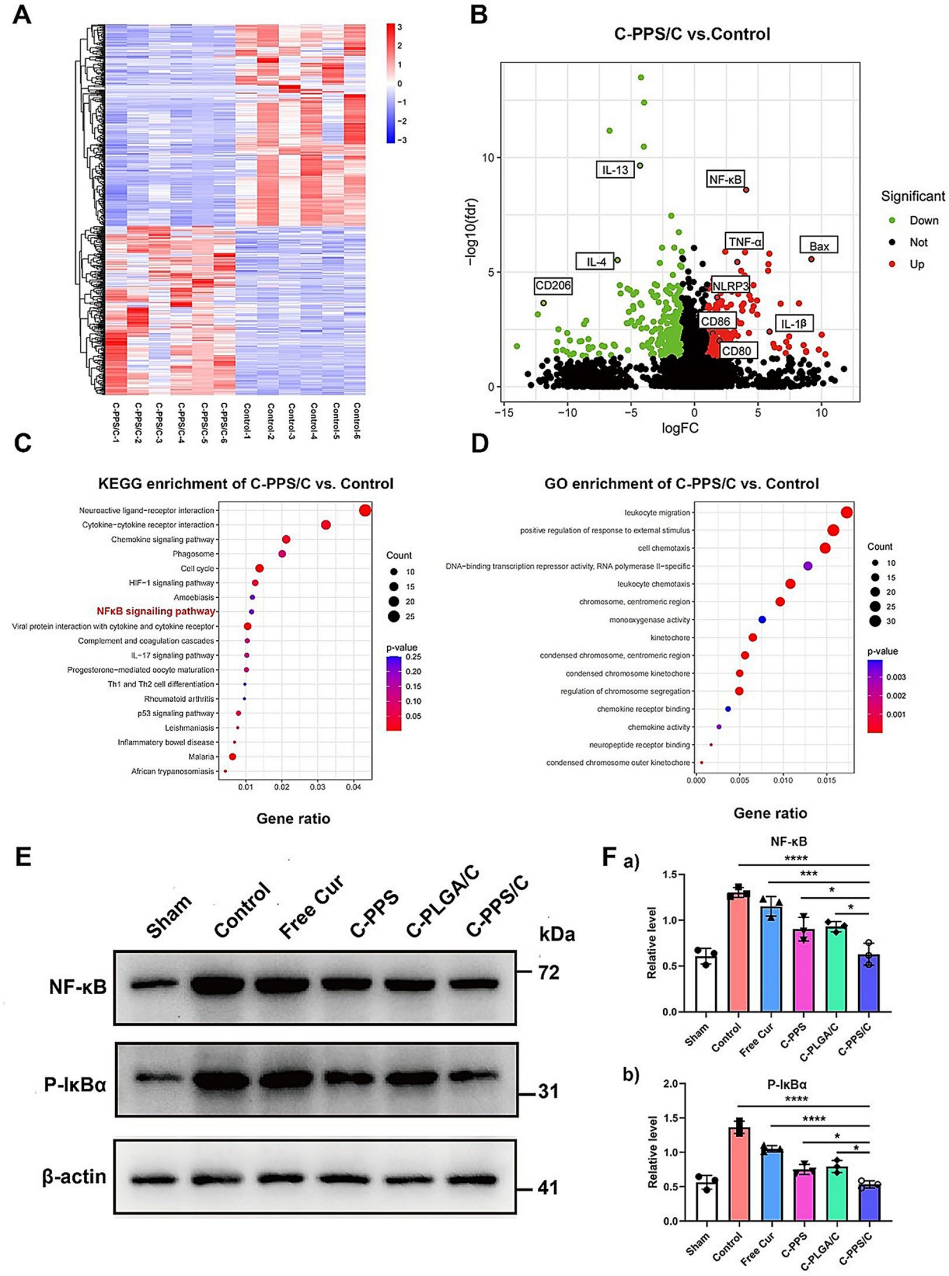
RNA-sequencing analysis of inflammation changes following C-PPS/C treatment. *n* = 6. (**A**) C-PPS/C, and Control groups’ gene heatmaps. (**B**) Analysis of differences in expression of genes between the C-PPS/C group and the Control group. (**C**) Enrichment analysis of KEGG pathway in the C-PPS/C group versus the Control group. (**D**) GO analysis in the C-PPS/C group and the Control group. C-PPS/C nanoparticles exert their effects by modulating the NF-κB signaling pathway. (**E**) Western blotting of NF-κB and P-IκBα in injured tissue. (**F**) Quantification of (a) NF-κB and (b) P-IκBα of each group. *n* = 3. Data are presented as the means ± SD. ^***^p < 0.001 and ^****^p < 0.0001

To further explore the mechanism by which C-PPS/C nanoparticles exert their effects on TBI, we conducted transcriptomic analysis on brain tissues before and after treatment. A heatmap of differentially expressed genes (DEGs) revealed significant differences between the two groups (Fig. 7A). Using|log fold change| > 1 and p-value < 0.05 as the screening thresholds, a total of 658 differentially expressed genes were detected in the brain tissue of mice treated with C-PPS/C nanoparticles compared to the Control group. The top 10 DEGs in the brain tissues of mice treated with C-PPS/C nanoparticles compared with those in the Control group were all closely related to inflammation (Fig. 7B). Kyoto Encyclopedia of Genes and Genomes (KEGG) and Gene Ontology (GO) enrichment analyses of the DEGs revealed enrichment in inflammation-related pathways such as “KEGG: NF-κB signaling pathway” and “GO: Leukocyte migration” (Fig. 7C-D). These findings suggest that the mechanism by which C-PPS/C nanoparticles exert their effects on TBI is closely associated with the suppression of inflammation.

### C-PPS/C nanoparticles exert their effects by modulating the NF-κB signaling pathway

RNA sequencing analysis revealed significant changes in the NF-κB signaling pathway in brain tissue from TBI mice treated with C-PPS/C. Following TBI, the NF-κB pathway plays a critical role in secondary brain injury. As a key transcription factor, NF-κB is activated post-injury, regulating the expression of numerous genes related to immune and inflammatory responses [35]. Its activation is often accompanied by oxidative stress and the release of inflammatory factors, which exacerbate neuroinflammation and contribute to secondary brain damage [36]. Phospho-IκBα (P-IκBα) is a pivotal molecule in NF-κB pathway regulation. Under resting conditions, NF-κB binds with IκBα and remains sequestered in the cytoplasm, preventing gene activation. Upon stimulation (such as by inflammation or oxidative stress), IκBα is phosphorylated by IκB kinase, generating P-IκBα. P-IκBα is then ubiquitinated and degraded, releasing NF-κB to translocate to the nucleus, where it activates various pro-inflammatory and immune-related genes. As shown in Fig. 7E and F, C-PPS/C nanoparticles inhibited the excessive activation of NF-κB and P-IκBα following TBI, thus suppressing neuroinflammation and providing a protective effect (Fig. 7E Control vs. C-PPS/C, *p* < 0.0001, 95% CI: 0.4088 to 0.9385; Free Cur vs. C-PPS/C, *p* = 0.0003, 95% CI: 0.2572 to 0.7869; C-PPS vs. C-PPS/C, *p* = 0.0409, 95% CI: 0.009477 to 0.5392; C-PLGA/C vs. C-PPS/C, *p* = 0.0231, 95% CI: 0.03619 to 0.5659; Fig. 7F Control vs. C-PPS/C, *p* < 0.0001, 95% CI: 0.6179 to 1.042; Free Cur vs. C-PPS/C, *p* < 0.0001, 95% CI: 0.3040 to 0.7284; C-PPS vs. C-PPS/C, *p* = 0.0429, 95% CI: 0.005776 to 0.4301; C-PLGA/C vs. C-PPS/C, *p* = 0.0141, 95% CI: 0.04752 to 0.4719).

### C-PPS/C nanoparticles increase emotion and neurological function after TBI

**Fig. 8:**
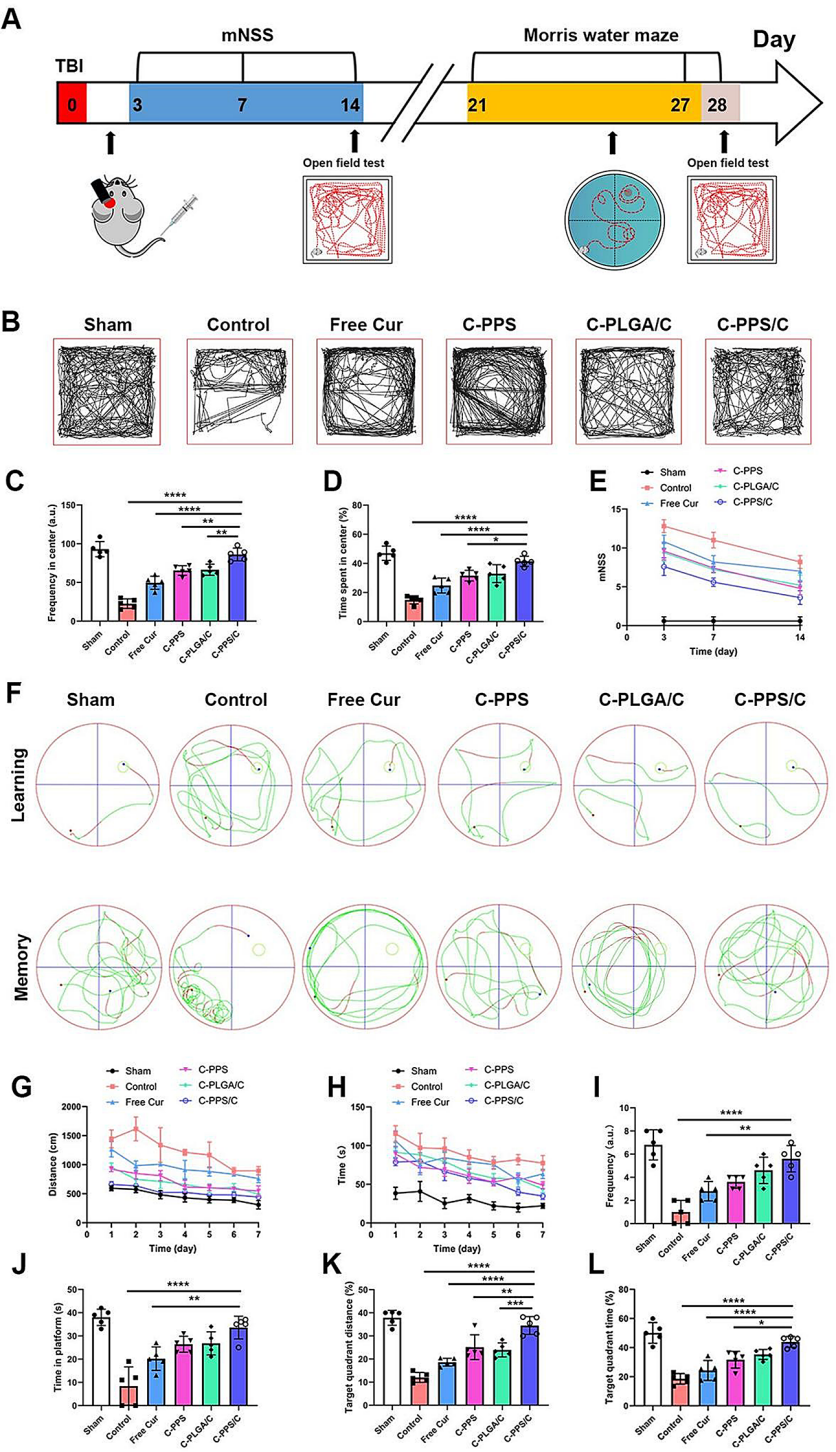
C-PPS/C nanoparticles increase emotion and neurological function after TBI. (**A**) mNSS, open field test, and Morris water maze analysis schedule. *n* = 5. (**B**) Representative images of the open field test results of each group on Day 14 after TBI. (**C**) Frequency in center on Day 14 after TBI. (**D**) Time spent in center on Day 14 after TBI. (**E**) mNSSs were calculated on Day 3, 7, and 14 after TBI. (**F**) Representative images of the Morris water maze of each group during the learning and memory periods. (**G**) Swimming distance to the platform during the learning period, (**H**) searching time for the platform during the learning period, (**I**) frequency of being on the platform area, (**J**) time in platform, (**K**) target quadrant distance and (**L**) target quadrant time during the memory period. Data are presented as the means ± SDs, ^*^p < 0.05, ^**^p < 0.01, ^***^p < 0.001 and ^****^p < 0.0001

Improving the loss of neurological function caused by TBI is of significant clinical importance. TBI not only results in the loss of neurological function but also profoundly impacts emotional and psychological health. In our study, we assessed the motor, emotional, learning, and memory abilities of mice following TBI. The diagram and schedule are shown in Fig. 8A.

Anxiety is a common emotional issue in TBI, where hippocampal damage and neuroinflammation disrupt the brain’s microenvironment, impairing the regulation of emotional and stress responses and increasing the risk of anxiety. In the open field test, we observed that mice exhibited significant anxiety after TBI, with reduced activity and reluctance to explore more areas of the field. The mice treated with C-PPS/C nanoparticles demonstrated the most significant reduction in anxiety, with behavior more closely resembling that of the Sham group (Fig. 8B-D) (Fig. 8C Control vs. C-PPS/C, *p* < 0.0001, 95% CI: -78.82 to -48.38; Free Cur vs. C-PPS/C, *p* < 0.0001, 95% CI: -51.82 to -21.38; C-PPS vs. C-PPS/C, *p* = 0.0036, 95% CI: -36.02 to -5.581; C-PLGA/C vs. C-PPS/C, *p* = 0.0059, 95% CI: -35.02 to -4.581; Fig. 8D Control vs. C-PPS/C, *p* < 0.0001, 95% CI: -35.32 to -17.74; Free Cur vs. C-PPS/C, *p* < 0.0001, 95% CI: -25.46 to -7.881; C-PPS vs. C-PPS/C, *p* = 0.0213, 95% CI: -18.67 to -1.091; C-PLGA/C vs. C-PPS/C, *p* = 0.0609, 95% CI: -17.32 to 0.2630). To further assess the impact of nanoparticles on long-term emotional and neurological functions, we conducted an open field test on day 28 post-injury in mice from different treatment groups (Fig. S5). Over time, anxiety levels improved in all groups, with the TBI mice treated with C-PPS/C nanoparticles showing more significant recovery. These findings suggest that C-PPS/C nanoparticles can, to some extent, alleviate mid- and long-term anxiety and neurological deficits in TBI mice.

The modified neurological severity score (mNSS) is a commonly used system for evaluating neurological impairment in mice after TBI, where higher scores indicate more severe functional damage [37]. We assessed the scores at 3, 7, and 14 days post-TBI in each group and found that the mice in the C-PPS/C group exhibited significant improvement in neurological function (Fig. 8E).

The Morris water maze is a widely used behavioral test to evaluate spatial learning and memory in mice [38, 39]. On the 21st day post-injury, mice were placed in a large pool and encouraged to find a clearly visible platform. Over the following 7 days (Days 21–27), the mice underwent trials in the same pool. Finally, on Day 28, the platform was removed, and the paths of the mice were assessed (memory phase). By recording the differences in the ability of the mice to locate the platform during the learning and memory phases, we assessed the spatial learning and memory capabilities of each group (Fig. 8F). As shown in Fig. 8G and H, during the learning phase, the C-PPS, C-PLGA/C, and C-PPS/C groups of mice spent less time and covered shorter distances to find the platform than the Control and Free Cur groups. In the memory phase, the C-PPS/C group exhibited significant improvements in various metrics, including frequency and time spent in the platform area, distance traveled in the target quadrant, and time spent in the target quadrant (Fig. 8I-L) (Fig. 8I Control vs. C-PPS/C, *p* < 0.0001, 95% CI: -6.604 to -2.596; Free Cur vs. C-PPS/C, *p* = 0.0028, 95% CI: -4.804 to -0.7962; C-PPS vs. C-PPS/C, *p* = 0.0506, 95% CI: -4.004 to 0.0038; C-PLGA/C vs. C-PPS/C, *p* = 0.6413, 95% CI: -3.004 to 1.004; Fig. 8J Control vs. C-PPS/C, *p* < 0.0001, 95% CI: -35.51 to -14.89; Free Cur vs. C-PPS/C, *p* = 0.0059, 95% CI: -23.71 to -3.089; C-PPS vs. C-PPS/C, *p* = 0.2925, 95% CI: -17.51 to 3.111; C-PLGA/C vs. C-PPS/C, *p* = 0.3509, 95% CI: -17.11 to 3.511; Fig. 8K Control vs. C-PPS/C, *p* < 0.0001, 95% CI: -29.16 to -15.75; Free Cur vs. C-PPS/C, *p* < 0.0001, 95% CI: -22.57 to -9.161; C-PPS vs. C-PPS/C, *p* = 0.0028, 95% CI: -16.08 to -2.667; C-PLGA/C vs. C-PPS/C, *p* = 0.0008, 95% CI: -17.24 to -3.827; Fig. 8L Control vs. C-PPS/C, *p* < 0.0001, 95% CI: -35.76 to -14.96; Free Cur vs. C-PPS/C, *p* < 0.0001, 95% CI: -30.01 to -9.214; C-PPS vs. C-PPS/C, *p* = 0.0147, 95% CI: -22.63 to -1.828; C-PLGA/C vs. C-PPS/C, *p* = 0.1446, 95% CI: -19.03 to 1.766).

## Materials and methods

### Synthesis and characterization of PPS120

In a dry and nitrogen-purged 50 mL round-bottom flask, 1,8-diazabicyclo [5.4.0] undec-7-ene (DBU) (3 mmol, 0.448 mL) was dissolved in 15 mL of dry tetrahydrofuran and degassed for 30 min. The reaction mixture was then cooled to 0 ℃. A 30-minute degassed solution of 1-butane thiol (1.0 mmol, 0.070 mL) in 10 mL tetrahydrofuran was added dropwise to this flask, and the mixture was allowed to react for 30 min. Subsequently, freshly distilled and degassed propylene sulfide (60 mmol, 4.68 mL) was added to the reaction mixture, and the temperature was maintained at 0 ℃ for 2 h. The reaction was quenched by adding 2-iodoethanol (2 mmol, 0.37 g) and stirring overnight at room temperature. The following day, the polymer mixture was filtered to remove precipitated salts and further purified by three rounds of precipitation cold methanol. Finally, the product was vacuum-dried to yield a colorless viscous polymer, PPS_120_ (3.8 g, 85.6%). ^1^H NMR was used to verify that the synthesis was successful.

### Preparation of the nanoparticles

PPS_120_ (1.0 mg), lecithin (0.1 mg), DSPE-PEG2000-CAQK (0.06 mg), DSPE-PEG2000-MAL (0.04 mg), and Cur (0.12 mg) were dissolved in dimethyl sulfoxide (200 µL) as the solvent. The mixture was then added dropwise to PBS (800 µL) and allowed to react for 10 min, followed by gentle stirring at room temperature for approximately 2 h to form C-PPS/C nanoparticles. The free Cur and DMSO were removed by purifying C-PPS/C nanoparticles. After dialysis in PBS (pH 7.4) for 12 h, C-PPS/C nanoparticles was stored at 4 °C. The synthesis methods for C-PPS nanoparticles and C-PLGA/C nanoparticles were the same as for C-PPS/C nanoparticles.

### Characterization of the nanoparticles

The particle size and zeta potential were measured using a laser particle size analyzer (Zetasizer NanoZS90, Malvern, UK). The morphology of the nanoparticles was observed using TEM (Tecnai G2 Spirit, FEI, USA). The amount of Cur was assayed by HPLC (Waters e2695, USA) using a C18 (250 × 4.6 mm) column, with the acetonitrile: water (60:40) mobile phase run at a flow rate of 1 ml/min. The temperature was 30 °C and the sample size was 20 µL. The Cur’s loading efficiency (%) and content (%) were calculated as follows:

$$\begin{gathered}\:Loading\:efficiency\:((\% )\: = \hfill \\\:(weight\:of\:drug\:loaded) \hfill \\\:/\:(weight\:of\:drug\:in\:feed) \hfill \\\times 100\% \: = \:7.15\% \hfill \\ \end{gathered} $$

$$\begin{gathered} Loading\,content\left( \% \right) \hfill \\= \left( {weight\,of\,drug\,loaded} \right)\: \hfill \\/\left( {weight\,of\,nanoparticles} \right) \hfill \\\times 100\% = 77\% \hfill \\ \end{gathered} $$

One milliliter of C-PPS/C nanoparticles or C-PLGA/C nanoparticles was transferred into a dialysis bag and immersed in 100 mL of PBS or PBS containing H_2_O_2_ (1mM/mL) respectively. The samples were maintained at 37 °C, and 200 µL samples were collected at different time intervals, with an equivalent volume of PBS added to maintain the volume. The drug release efficiency of Cur was analyzed using HPLC.

### Cell culture

HA1800 and BV2 cells (sourced from the Cell Bank of the Chinese Academy of Sciences, Shanghai) were grown in Dulbecco’s modified Eagle medium containing 1% penicillin-streptomycin and 10% fetal bovine serum. The cells were incubated in a humidified atmosphere at 37 °C with 5% CO_2_.

### In vitro ROS detection

ROS detection kits (Beyotime, Shanghai, China) were used to measure intracellular ROS levels following the manufacturer’s protocol. The cells were seeded in 96-well plates at a density of 1 × 10⁵ cells per well and co-incubated with 10 µl of nanoparticles (C-PPS, C-PLGA/C, or C-PPS/C) and hydrogen peroxide (H_2_O_2_, 0.5 mM or 0.25 mM) for 2 h. The Control group remained untreated. Subsequently, cells were stained with 10 µM dichlorofluorescein diacetate (DCFH-DA; Beyotime, Shanghai, China) in serum-free medium at 37 °C for 30 min. After removing DCFH-DA, the generation of ROS inside the cells was visualized using fluorescence microscopy (excitation 488 nm; emission 519 nm), and the fluorescence intensity was quantified using ImageJ software. Subsequently, the fluorescence of DCFH-DA in each group was quantitatively assessed using flow cytometry, and the data were analyzed with FlowJo software (Becton, Dickinson & Company, USA).

### Animals and TBI model protocol

The animals were obtained from Jiangsu Jicui Yaokang Biotechnology Co., Ltd., with certificate number B202404220463. All animal experiments complied with the ARRIVE guidelines and were carried out in accordance with the National Research Council’s Guide for the Care and Use of Laboratory Animals. The Institutional Animal Care and Use Committee approval (IACUC) number was No.137. Male ICR mice (5 weeks old) were anesthetized with isoflurane and secured in a stereotaxic frame. Under sterile conditions, the skull was fully exposed without damaging the dura mater, and the bone was removed. A controlled cortical impact (CCI) TBI model was established using an electromagnetically controlled impact device (PinPoint™ PCI3000 Precision Cortical Impactor, Hatteras Instruments, Cary, NC, USA). The tip of a 3 mm impactor piston was positioned perpendicular to the exposed cortical surface to create a severe traumatic brain injury model [40]. The CCI parameters were as follows: impact speed of 3.5 m/s, deformation depth of 2.5 mm, and duration of 400 ms. Under these conditions, no mouse mortality was observed post-TBI. Severe TBI is characterized by the loss of hippocampal and cortical tissue. The successful establishment of the severe TBI model was confirmed by H&E staining of mouse brain sections. In the Sham group, the mice underwent craniectomy but did not receive any affected. Free Cur, PBS, C-PPS/C nanoparticles, C-PLGA/C nanoparticles and C-PPS/C nanoparticles were administered. The drugs were administered via tail vein 6 h after TBI at a dose based on 10 mg/kg of Cur. The Sham group received no treatment.

### Tissue targeting distribution analysis

Dir-PPS/C nanoparticles and Dir-C-PPS/C nanoparticles were injected into the mice. At 6 and 24 h post-injection, the in vivo distribution of the nanoparticles was observed using a small animal imaging system (Caliper, USA).

### Biocompatibility evaluation in vivo

To evaluate the biosafety of the nanoparticles, organ and blood samples were collected from each group of mice on the 7th day post-administration. The collected organs included the heart, liver, spleen, lungs, and kidneys. Blood samples were used to measure AST, ALT, BUN, and CREA levels to assess liver and kidney function. The organs were subjected to H&E staining and examined under a Leica microscope.

### MRI test

Three days post-TBI, changes in the brain water content of each group of mice were detected using a 7.0 T micro-MRI scanner (Bruker, BioSpec 70/20). The parameters were as follows: repetition time = 2500 ms, echo time = 36 ms, slice thickness = 1 mm, field of view = 20 × 20 mm, and image matrix = 256 × 256. The brain edema volume at each time point was calculated using ImageJ software.

### Brain water content test

Three days post-TBI, the injured hemispheres of the mouse brains were isolated and their wet weights were measured. The tissues were then dried at 80 °C for 72 h. The brain water content was calculated using the following formula: Brain water content = (wet weight - dry weight) / wet weight × 100%.

### Evans blue assay

On the 3rd day post-TBI, 2 ml/kg of Evans blue (Aladdin, Shanghai, China) was intravenously injected into each group of mice, which were allowed to circulate for 3 h. The mice were then perfused with saline, and their brains were removed, photographed, and weighed. The injured hemispheres were thoroughly mixed and homogenized with 4 ml of 1 M potassium hydroxide. One milliliter of homogenate was combined with 5 ml of 5:13 0.2 mmol phosphoric acid: acetone, and the resulting mixture was centrifuged at 3000 × g for 30 min. The absorbance of the supernatant was measured at 620 nm.

### Detection of oxidative stress in vivo

On the 3rd day post-injury, each mouse was administered an intraperitoneal injection of DHE at a dose of 6 µg/g body weight. One hour after DHE injection, 5 mice from each group were sacrificed, and their brain tissues were quickly isolated. The tissue samples were rinsed in ice-cold PBS to remove any blood. The tissues were then prepared into as single-cell suspensions, and the fluorescence intensity was measured. DHE reacts with ROS to emit fluorescence (excitation wavelength of 488 nm, emission wavelength of 585 nm). Three days post-TBI, the injured hemispheres of 5 mice from each group were isolated and weighed, and the isolation process conducted on ice. Lysis buffer was added to the tissue at a ratio of 1:10, and the mixture was allowed to react for 15 min. After centrifugation at 12,000 × g for 10 min, the supernatant was collected. The levels of SOD, GSH-Px, and MDA were measured using enzyme-linked immunosorbent assay kits (Bosters Biological Technology Co., Ltd., Wuhan, China).

### Flow cytometry analysis

To assess the effects of various treatments on macrophage repolarization from the M1 to the M2 phenotype, flow cytometry was used. The treated mice from each group were euthanized on Day 3. The perfused brain tissues were collected and digested in 1 mg mL-1 collagenase IV or, 200 µg mL-1 DNase I in 10 mM HEPES buffer at 37 °C for 60 min to obtain a cell suspension. The dissociated cells were subsequently filtered through a 70 μm nylon cell strainer, and red blood cells were removed with RBC lysis buffer (3 mL, Biolegend) for 5 min at room temperature. Single-cell suspensions were stained with relevant antibodies (anti-F4/80-FITC, anti-CD11b-PE, anti-CD206-APC) for 30 min at 4 °C. The cells were measured with a flow cytometer (BD Facs Canto II, USA) and analyzed using FlowJo (V10) software.

### Quantification of inflammatory response

The brain tissue was isolated on Day 3, and the tissue suspension was prepared as previously described. The levels of IL-1β, IL-6, TNF-α, and CXCL-1 were quantified using enzyme-linked immunosorbent assay kits from Bosters Biological Technology Co., Ltd., Wuhan, China.

### Staining of brain tissue sections

On the 7th day, 3 mice from each group were sacrificed, and their brain tissues were harvested for histological analysis. The tissues were fixed in 10% formalin, embedded in paraffin, and then sectioned into 20 μm slices using a paraffin microtome. These sections were subsequently subjected to H&E, Nissl, TUNEL, Iba-1, and GFAP staining. Observations were made under a microscope, using visible light for H&E, Nissl, and GFAP staining, and fluorescent light for TUNEL staining.

### RNA-sequencing

On the 3rd day following TBI, brain tissue samples from the injury site were sent to Genefund Biotech (Shanghai, China) for RNA-seq library preparation. After clustering, transcriptome sequencing was performed on the DNBSEQ-T7 platform, generating raw reads. Adaptor sequences, ambiguous “N” nucleotides (where the proportion of “N” exceeded 5%), and low-quality sequences (with a quality score below 10) were removed before data quality was assessed using the FastQC tool. The ggplotR package was employed to identify StringTie genes, with a|log2 fold change| ≥ 1 or a *p*-value < 0.05 set as the threshold for statistical significance.

### Western blotting

On the 3rd day following TBI, brain tissue samples from the injury site were detected by western blotting. The primary antibodies used were as follows: anti-NF-κB p65 (Abcam, UK) and P-IκBα (Ser32, Ser36) monoclonal antibody (Cell Signaling technology, USA). The secondary antibody was sourced from ABclonal Biotechnology Co., Ltd. The intensity of the bands was analyzed using ImageJ software, while protein concentration was measured via the bicinchoninic acid assay.

### Emotional and neurological function assessment

On Day 3, 7, and 14 post-TBI, the motor, sensory, reflex, and balance abilities of 5 mice in each group were evaluated using the mNSS. On Day 14 and Day 28, anxiety levels in the mice were assessed using the open field test (SANS SA215, China). The frequency of entries into the central area and the ratio of time spent in the central area to the total movement time were recorded as indicators of anxiety. The spatial learning and memory abilities of the mice were evaluated using the Morris water maze test (SANS SA201, China). Over a period of 7 days (Days 21–27), the mice underwent 5 trials each day to find the hidden platform. On Day 28, the platform was removed, and the mice were observed for 1 min to evaluate their search paths. All data collected during the tests were recorded and analyzed using a video tracking system.

### Statistical analysis

All statistical analyses were conducted using IBM SPSS Statistics for Windows, version 16.0 (IBM Corp, USA), and all plots were generated with GraphPad Prism version 10.0 (GraphPad Software, USA). The data are presented as means ± standard deviations (SDs). Statistical significance was determined using one-way analysis of variance (ANOVA) followed by Tukey’s post hoc test, and repeated measures one-way ANOVA with Bonferroni correction. A p-value of *p < 0.05, **p < 0.01, ***p < 0.001, and ****p < 0.0001 was considered statistically significant.

## Discussion

TBI is a prevalent type of traumatic injury that imposes significant economic burdens on patients’ families and society, and is one of the leading causes of disability and death worldwide [1, 2]. TBI is associated with high mortality rate, and even survivors may face long-term disabilities, including cognitive, motor, and behavioral impairments [41]. Currently, effective targeted therapies for secondary brain injury, which is a crucial phase in TBI treatment. Post-TBI, disturbances in the oxygen supply and a surge in oxidative stress lead to the production of excessive ROS, which exacerbates neuroinflammation. Furthermore, a large number of inflammatory cells in the cranial cavity after TBI promote the generation of ROS, creating a vicious cycle of “ROS-neuroinflammation”. Prompt ROS scavenging and anti-inflammatory interventions are essential to breaking this harmful cycle and facilitating the repair of organ damage [16]. Therefore, breaking this “ROS-neuroinflammation” cycle and regulating the oxidative and inflammatory microenvironment are key to addressing secondary brain injury following TBI [11, 42].

The potent antioxidant and anti-inflammatory properties of Cur suggest its potential as a therapeutic agent for TBI. Increasing research has highlighted the value of Cur in TBI treatment [43, 44]. However, the clinical application of Cur in TBI has progressed slowly due to several limitations: (i) Low Solubility and solubility and absorption: Cur has extremely low solubility in water, which restricts its distribution and absorption in the body. The oral absorption of Cur is poor, resulting in low plasma concentrations that make it difficult to achieve effective therapeutic doses. Additionally, Cur is rapidly metabolized in the body and has a short half-life. (ii) Chemical Instability: Cur is chemically unstable, and degrades under light, pH changes, and high temperatures, which affects its storage and usage. Its strong reducing properties also make it susceptible to oxidation, leading to decreased activity. (iii) Poor tissue penetration: Cur has difficulty effectively penetrating target tissues or organs, particularly the central nervous system, due to the presence of the BBB, which limits its therapeutic efficacy. Inspired by the ROS-scavenging capabilities of thioethers in PPS, we synthesized PPS_120_ (a polymer with a degree of polymerization of 120) as the core to encapsulate Cur. We modified the surface with hydrophilic groups to address the instability of Cur in blood. Additionally, we functionalized the nanoparticle surface with CAQK to facilitate effective BBB penetration and accumulation in injured brain tissue. The PPS core responds to and consumes ROS, subsequently releasing Cur in the target area to exert neuroprotective effects. This strategy addresses the issues of the low bioavailability and lack of targeting of Cur. Therefore, in this study, we designed and prepared C-PPS/C nanoparticles, leveraging the synergistic advantages of PPS and Cur to overcome the vicious “ROS-neuroinflammation” cycle after TBI, thereby providing neuroprotection.

In this study, we prepared C-PPS/C nanoparticles with a uniform structure and good stability. These nanoparticles respond to and release Cur in high H_2_O_2_ environments, providing a prerequisite for subsequent experiments. We demonstrated both in vitro and in vivo that C-PPS/C nanoparticles effectively scavenge ROS, thereby inhibiting oxidative stress. More importantly, we found that C-PPS/C nanoparticles enhance the endogenous antioxidant capacity by increasing the activity of intracellular antioxidant enzymes such as SOD and GSH-px, thereby increasing the antioxidant defense of neuronal cells. These results indicate that C-PPS/C nanoparticles exerts its effects through both exogenous ROS scavenging and endogenous antioxidant capacity.

Neuroinflammation is a key factor in the pathological process following TBI, initiating rapidly after injury and persisting for days to weeks, significantly affecting patient outcomes [45]. Following TBI, microglia are the first immune cells to be activated, transitioning from a resting state to an activated state, changing morphology, and releasing various inflammatory mediators. Astrocytes are also activated after TBI, releasing pro-inflammatory cytokines and ROS, further exacerbating inflammation and oxidative stress. Disruption of the BBB in TBI allows peripheral immune cells such as neutrophils, monocytes, and macrophages to infiltrate brain tissue, participating in the inflammatory response [46]. M2-type microglia play anti-inflammatory and reparative roles in TBI, aiding in neural regeneration and functional recovery. Cur can promote the polarization of microglia towards the M2 phenotype. Experiments in vivo confirmed that C-PPS/C nanoparticles promote microglial polarization to the M2 phenotype, thereby exerting anti-inflammatory effects. C-PPS/C nanoparticles reduced the levels of pro-inflammatory cytokines and chemokines in the brain tissue post-TBI, demonstrating its anti-inflammatory effects. Furthermore, Iba-1 and GFAP staining revealed that C-PPS/C nanoparticle inhibited the expression of pro-inflammatory microglia and astrocytes after TBI. Therefore, C-PPS/C nanoparticles have both antioxidant and anti-inflammatory capabilities.

Brain edema is a common manifestation of secondary injury following TBI and poses a significant challenge in acute TBI management. This condition is directly associated with disruption of the BBB. TBI can directly damage the BBB, while excessive oxidative stress and inflammatory responses further compromise its integrity. The resulting increase in BBB permeability allows more fluids and inflammatory substances to infiltrate the brain tissue, exacerbating brain edema and creating a vicious cycle [26]. Therefore, fundamentally inhibiting oxidative stress and inflammation post-TBI and protecting the BBB are crucial for treating brain edema. We discovered that C-PPS/C mitigates BBB disruption following TBI, thereby reducing brain tissue edema. Tissue staining revealed that TBI mice treated with C-PPS/C nanoparticles reduced neuronal death and apoptosis, as well as less tissue damage. These findings indicate that C-PPS/C nanoparticles can alleviate acute brain edema and exert neuroprotective effects.

Through RNA-sequencing analysis, we observed significant changes in inflammation-related pathways in mice treated with C-PPS/C nanoparticles, indicating its strong anti-inflammatory effects. We specifically focused on the NF-κB pathway, as NF-κB is a transcription factor that regulates the expression of numerous genes involved in immune and inflammatory responses [35]. In the context of TBI, excessive ROS production triggers oxidative stress, leading to the activation of the NF-κB pathway [47]. Once activated, NF-κB induces the expression of pro-apoptotic genes, promoting neuronal death. Additionally, NF-κB activation initiates an inflammatory cascade, driving the production of pro-inflammatory cytokines such as TNF-α, IL-1β, and IL-6, which further exacerbates brain tissue damage by increasing inflammation and immune cell infiltration [36, 48]. As such, NF-κB is the key regulator in the vicious cycle of “ROS-induced neuroinflammation” following TBI. Our exploration and analysis of NF-κB as a key molecular target in TBI will contribute to a deeper understanding of the mechanisms underlying TBI progression. Cur, delivered by C-PPS/C nanoparticles, is the key active ingredient, and its interaction with the NF-κB signaling pathway has garnered significant attention. As a critical transcription factor in inflammatory responses, NF-κB plays a central role in the progression of TBI. Its activation triggers a cascade of inflammatory reactions, exacerbating neuronal damage and death. Previous studies have shown that Cur significantly inhibits NF-κB signaling. Cur can directly suppress the activation of NF-κB, microglia, and astrocytes, downregulate the expression of pro-inflammatory cytokines TNF-α and IL-1β, and protect damaged neurons [49, 50]. Additionally, Cur can interfere with the early activation of the NF-κB signaling pathway by blocking the nuclear translocation of the NF-κB subunit p65, inhibiting endoplasmic reticulum stress and neuroinflammation, and improving cognitive function in mice [51]. Cur also regulates multiple NF-κB-related signaling pathways. For example, it upregulates PPARγ expression and modulates microRNAs such as miR-199b-5p to influence the IKKβ/NF-κB pathway, thereby indirectly inhibiting NF-κB signaling [52]. By targeting the delivery of Cur to the injury site in TBI, C-PPS/C nanoparticles effectively inhibit NF-κB signaling post-TBI. However, in this study, we have only identified that C-PPS/C nanoparticles inhibit NF-κB signaling without providing a detailed analysis and proof of the underlying mechanisms. We plan to further explore and elucidate the relevant molecular mechanisms in future studies.

Patients with TBI often suffer from anxiety, as well as declines in cognitive and motor abilities, which significantly impact their quality of life and long-term prognosis. Neuroinflammation is considered a key mechanism in the development of anxiety [53]. The pro-inflammatory states of microglia and astrocytes, through the release of inflammatory cytokines, affect neural circuitry, leading to anxious behaviors. Persistent neuroinflammation and oxidative stress can cause cell damage, particularly in the hippocampus, impairing memory and learning abilities. Given the anti-inflammatory and antioxidant properties of C-PPS/C nanoparticles, we further explored their potential to improve behavioral outcomes in TBI mice. Our findings suggest that TBI mice treated with C-PPS/C nanoparticles exhibited some improvement in emotional, motor, cognitive, and learning functions. These findings suggest that C-PPS/C nanoparticles may have potential in the acute management of TBI and could influence long-term outcomes. However, further investigation is needed to better understand the relationship between C-PPS/C nanoparticles and behavioral improvements, as well as its clinical applicability.

Given the complex pathophysiological mechanisms of TBI, the duration and intensity of oxidative stress and inflammatory responses can vary significantly among individuals, highlighting the need for personalized treatment approaches. Clinical management must account for factors such as trauma type, severity, and patient-specific characteristics. Our design of C-PPS/C nanoparticles aims to offer new insights into the clinical treatment of TBI, but further research is essential to determine the optimal timing for intervention and develop tailored treatment strategies. Additionally, the intricate relationship between Cur, oxidative stress, inflammation, and neurological recovery post-TBI still requires deeper exploration of its underlying mechanisms. Continuous optimization of related materials is crucial to better align with clinical treatment needs. It is important to recognize the limitations of our current study and acknowledge the need for ongoing research to refine these approaches.

## Conclusion

In summary, this study designs ROS-scavenging C-PPS/C nanoparticles that synergize with Cur to treat TBI by breaking the “ROS-neuroinflammation” vicious cycle. The good targeting ability of C-PPS/C nanoparticles allows them to accumulate in the injured brain tissue after TBI, respond to and deplete excessive ROS, and concurrently release Cur. C-PPS/C nanoparticles disrupt the vicious cycle of “ROS-neuroinflammation” following TBI, protecting the BBB, reducing brain edema in the acute phase, and promoting long-term neurological recovery. These findings demonstrate their clinical application potential throughout the entire TBI treatment process, offering new insights for the clinical management of TBI.

## Electronic supplementary material

Below is the link to the electronic supplementary material.

Supplementary Material 1

## Data Availability

No datasets were generated or analysed during the current study.
