# Supplementary material for: Responsive nanoparticles synergize with Curcumin to break the “reactive oxygen Species-Neuroinflammation” vicious cycle, enhancing traumatic brain injury outcomes
Source: J Nanobiotechnology. 2025 Mar 5;23:172. doi: 10.1186/s12951-025-03251-y (PMC11881390; doi:10.1186/s12951-025-03251-y)
Supplement: Supplementary file 1 — Supplementary Material 1 [file 12951_2025_3251_MOESM1_ESM.docx]

**Responsive Nanoparticles Synergize with Curcumin to** **Break the “Reactive Oxygen Species-Neuroinflammation” Vicious Cycle, Enhancing Traumatic Brain Injury Outcomes**

Xianhua Fu^1,2#^, Yongkang Zhang^4#^, Jieguo Chen^5#^, Guangyao Mao^5^, Jiajia Tang^2^, Jin Xu^2^, Yuhan Han^3*^, Honglin Chen^2*^, Lianshu Ding^1*^

^1^ *Department of Neurosurgery, The Affiliated Huaian NO.1 People’s Hospital of Nanjing Medical University, Huaian, Jiangsu, China.*

^2^ *Department of Neurosurgery, The Affiliated Suqian First People’s Hospital of Nanjing Medical University, Suqian, China.*

^3^ *Brain Injury Center, Department of Neurosurgery，Ren Ji Hospital, Shanghai Jiao Tong University School of Medicine, Shanghai, China.*

^4^ *Department of Neurosurgery, The Affiliated Hospital of Xuzhou Medical University, Xuzhou, China*

^5^ *Clinical Laboratory, Affiliated Taizhou People’s Hospital of Nanjing Medical University, Taizhou, Jiangsu,China.*

*^*^* Corresponding author*.*

E-mail address: dlshu@njmu.edu.cn (L. Ding)；jsshychl@163.com (H. Chen)； hanyuhan1994@163.com (Y. Han)

^#^ These authors contributed equally to the present research.

**Supplementary material**


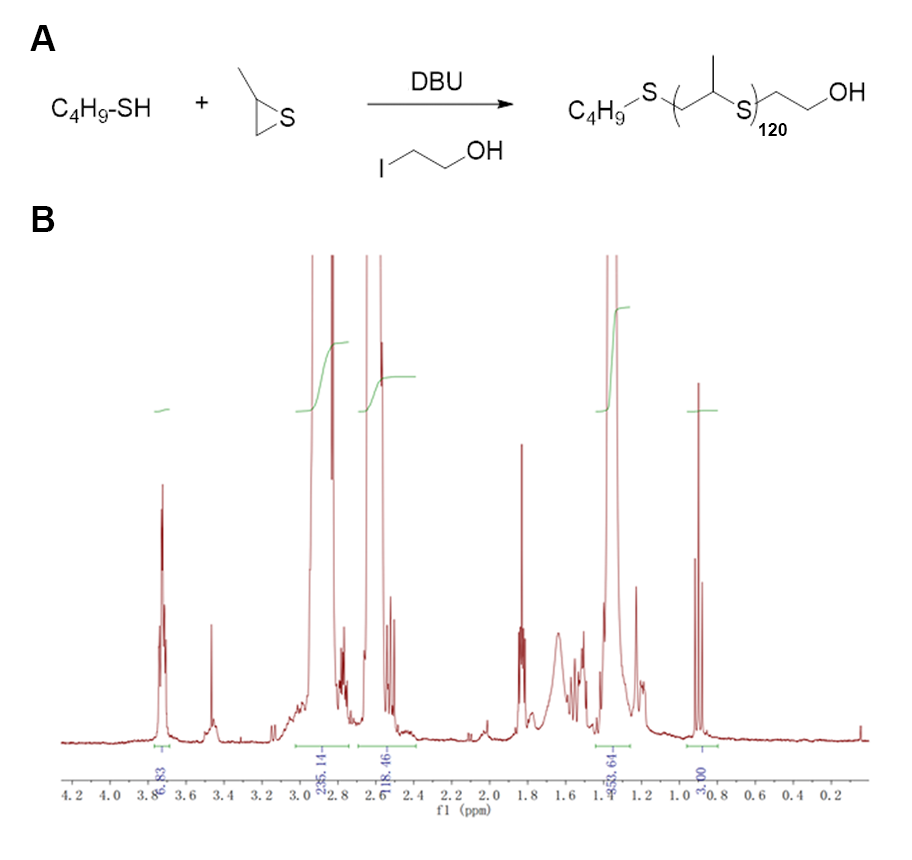


**Figure. S1** Synthesis and characteristics of PPS_120_. a) Synthesis route of PPS_120_. b) ^1^H NMR of PPS_120_.


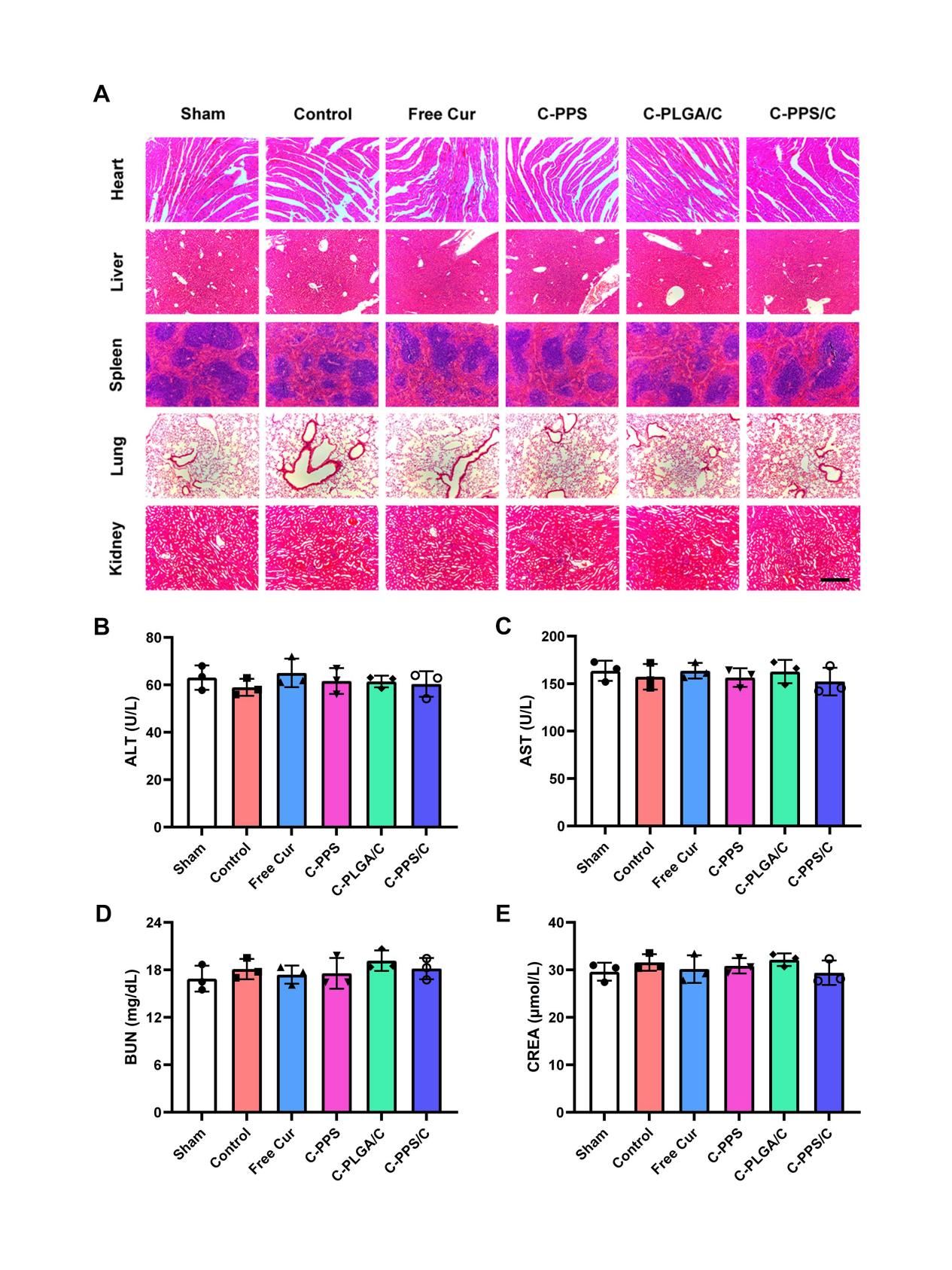


**Fig. S2** **C-PPS/C nanoparticles have good biocompatibility *in*** ***vivo*.** (A) H&E staining of the heart, liver, spleen, lung, and kidney of each grou. scale bar = 200 μm. (B) ALT, (C) AST, (D) BUN and (E) CREA levels in each group on the 7th day after TBI. n = 3. Data are presented as the means ± SDs.


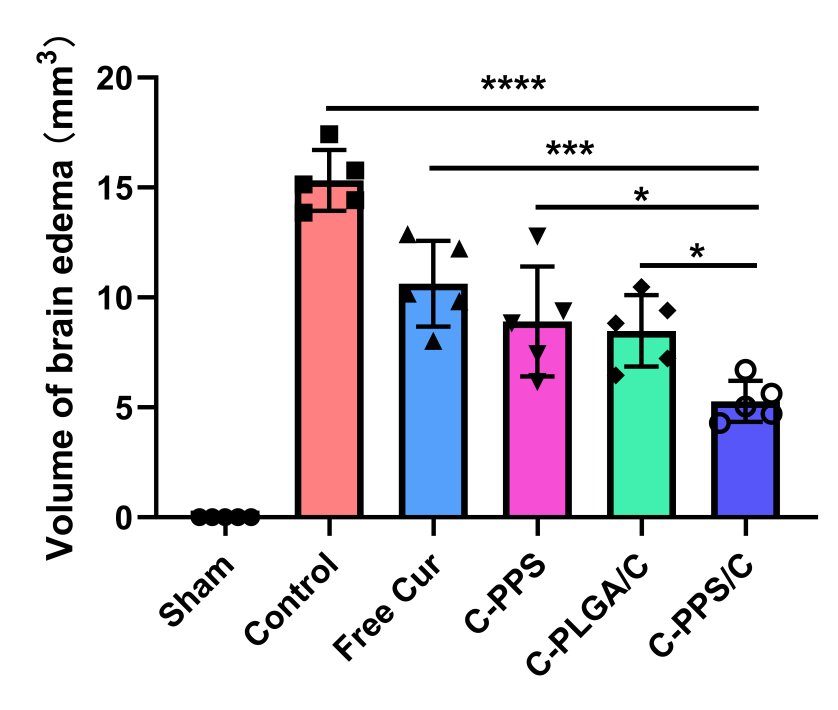


**Fig. S3** Quantification of the brain edema volume around the injured tissue on the 7th day after TBI. n = 5. Data are presented as the mean ± SD. ^*^*p* < 0.05, ^***^*p* < 0.001 and ^****^*p* < 0.0001.


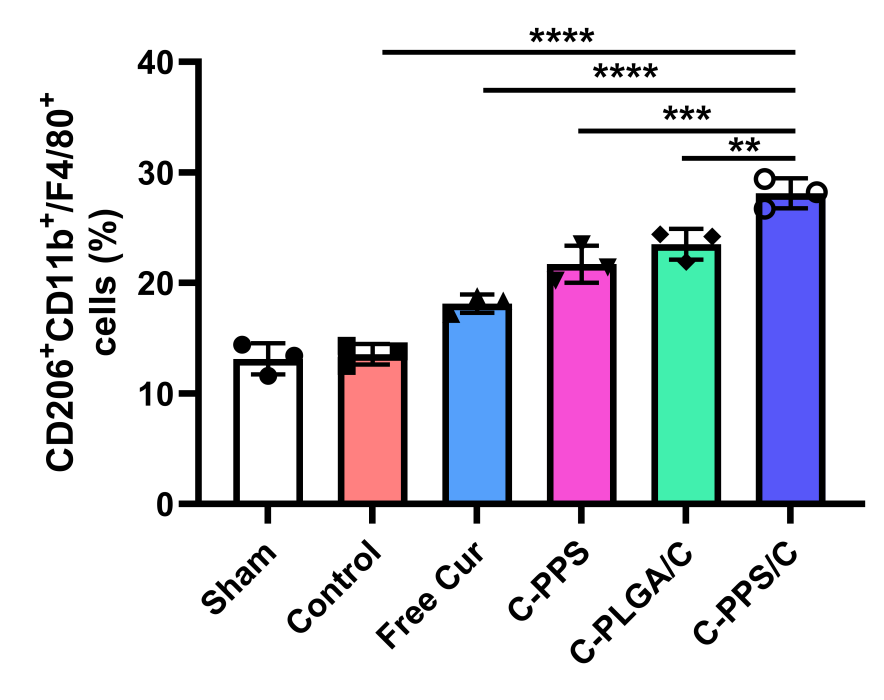


**Fig. S4** Quantification of macrophage type in brain tissue of each group. n = 3. Data are presented as the means ± SD. ^**^*p* < 0.01, ^***^*p* < 0.001 and ^****^*p* < 0.0001.


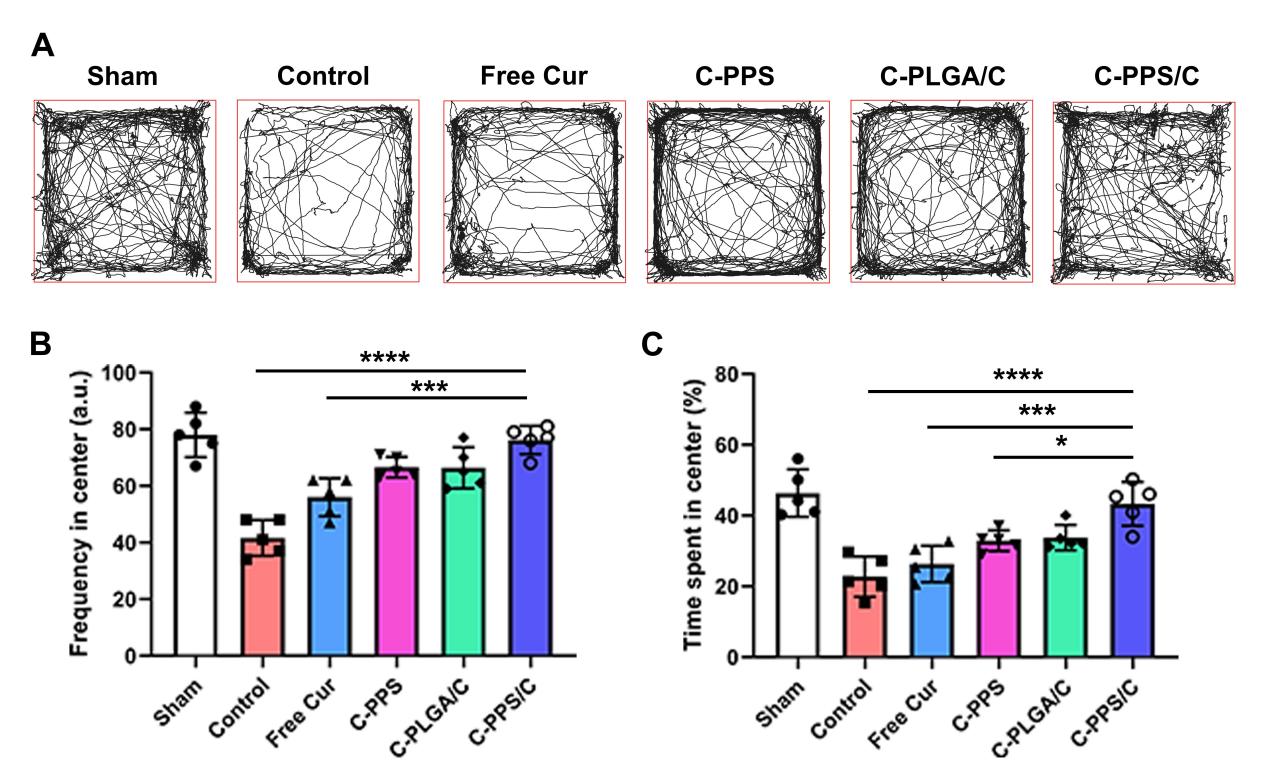


**Fig. S5** (A) Representative images of the open field test results of each group on Day 28 after TBI. (B) Frequency in center on Day 28 after TBI. (C) Time spent in center on Day 28 after TBI. n = 3. Data are presented as the means ± SD. ^*^*p* < 0.05, ^***^*p* < 0.001 and ^****^*p* < 0.0001.
